# LINC00607 facilitates endothelial VEGF-A receptor FLT1 splicing

**DOI:** 10.1016/j.ymthe.2026.03.038

**Published:** 2026-04-04

**Authors:** Frederike Lam, Timothy Warwick, James A. Oo, Agnes Y. Krüger, Nina-Naomi Kreis, Anastasiia Diagel, Judit Izquierdo Ponce, Praveenya Tirunagari, Marie E. Bayer, Olivia Nonn, Ralf Dechend, Thomas Walther, Reinier A. Boon, Andrew H. Baker, Stefan Günther, Ilka Wittig, Zhifen Chen, Michaela Müller-McNicoll, Frank Louwen, Ralf P. Brandes, Matthias S. Leisegang

**Affiliations:** 1 Goethe University, Institute for Cardiovascular Physiology, Frankfurt, Germany; 2 German Center of Cardiovascular Research (DZHK), Partner Site RheinMain, Frankfurt, Germany; 3 Goethe University, University Hospital Frankfurt, Division of Obstetrics and Prenatal Medicine, Frankfurt, Germany; 4 Technical University of Munich, Department of Cardiology, German Heart Centre Munich, School of Medicine and Health, Munich, Germany; 5 DZHK, Partner Site Munich Heart Alliance, Munich, Germany; 6 Charité - Universitätsmedizin Berlin, Corporate Member of Freie Universität Berlin and Humboldt-Universität zu Berlin, Berlin, Germany; 7 DZHK, Berlin, Germany; 8 HELIOS Klinikum Berlin-Buch, Department of Cardiology and Nephrology, Berlin, Germany; 9 Goethe University, University Hospital Frankfurt, Department of Cardiovascular Surgery, Frankfurt, Germany; 10 Goethe University, Institute for Cardiovascular Regeneration, Centre for Molecular Medicine, Frankfurt, Germany; 11 Department of Physiology, Amsterdam Cardiovascular Sciences, VU Medical Center, Amsterdam UMC, Amsterdam, the Netherlands; 12 Institute for Neuroscience and Cardiovascular Research, University of Edinburgh, Edinburgh, Scotland; 13 CARIM Institute, University of Maastricht, Maastricht, the Netherlands; 14 Max Planck Institute for Heart and Lung Research, Bad Nauheim, Germany; 15 Goethe University Frankfurt, Institute for Molecular Biosciences, Frankfurt, Germany; 16 Max Planck Institute for Biophysics, Frankfurt, Germany

**Keywords:** long non-coding RNA, angiogenesis, splicing, vascular endothelial growth factor, VEGF, LINC00607, preeclampsia, morpholino, sFLT1, soluble FMS-like tyrosine kinase-1

## Abstract

Angiogenesis is a key function of vascular endothelial cells and becomes aberrant in pathologies such as preeclampsia. An important mediator of angiogenesis is vascular endothelial growth factor (VEGF) receptor FLT1; however, alternative splicing of *FLT1* can generate soluble FLT1 (sFLT1), a decoy receptor that inhibits VEGF signaling. While some long non-coding RNAs (lncRNAs) are known to regulate splicing, their roles in endothelial biology remain poorly defined. Here, we identify lncRNA *LINC00607* as a critical regulator of FLT1 alternative splicing. Loss of *LINC00607* increased the formation of the anti-angiogenic sFLT1. CRISPR-mediated knockout of *LINC00607* promoted exon 15 inclusion in *FLT1*, elevating sFLT1 levels and blunting VEGF-driven angiogenesis—a defect reversed by sFLT1-neutralizing antibodies. *LINC00607* interacted with U2 small nuclear RNA (snRNA) to regulate exon 15 inclusion in *FLT1*, an interaction dependent on the chromatin-remodeler BRG1. A splice-blocking morpholino targeting the FLT1 intron14/exon15 junction specifically inhibited sFLT1 production by interacting with *LINC00607* and U2 snRNA, and its application increased VEGF-A-mediated sprouting. *LINC00607* expression inversely correlated with sFLT1 levels in vascular diseases. In preeclampsia, a multisystem pregnancy disorder involving hypertension and proteinuria, *LINC00607* was downregulated in early and late-stage preeclampsia compared with healthy pregnancies. *LINC00607* therefore fine-tunes VEGF signaling and might contribute to the pathophysiology of preeclampsia.

## Graphical abstract

**Figure:**
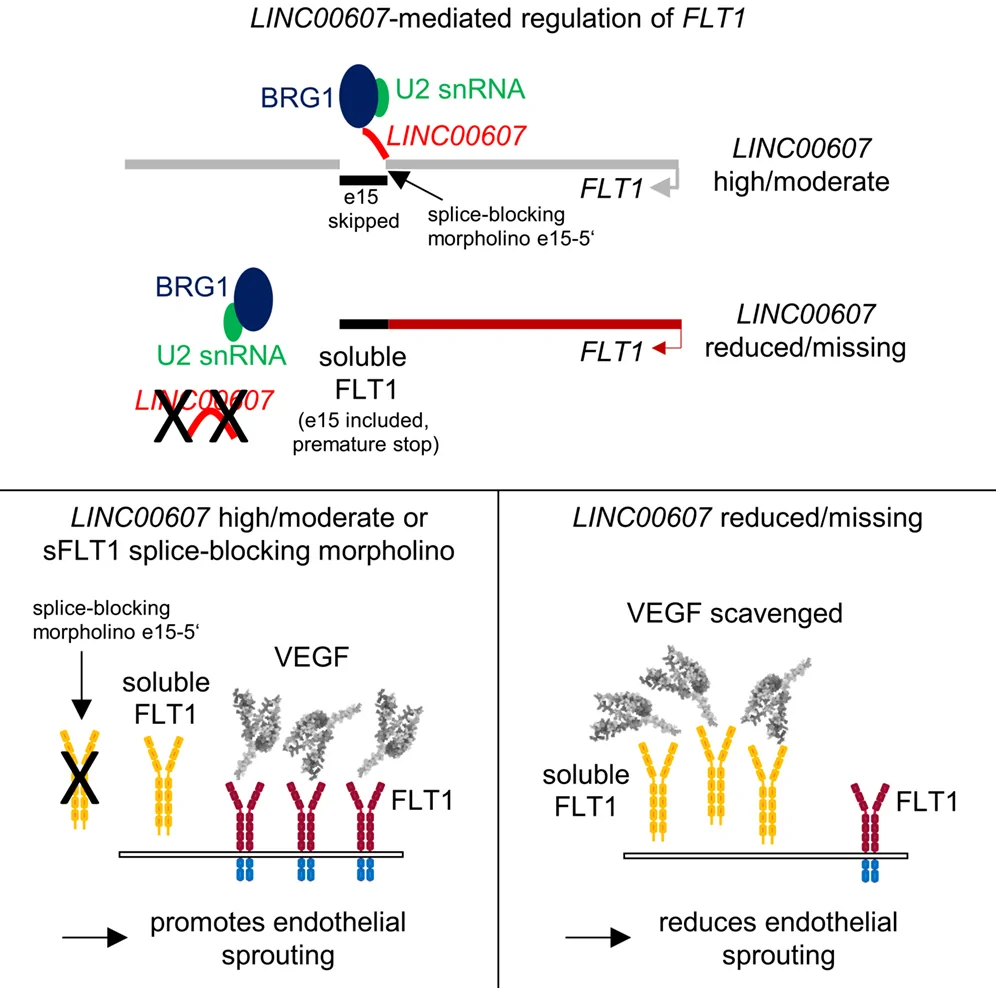

## Teaser

lncRNA *LINC00607* regulates vascular endothelial growth factor receptor FLT1 splicing to suppress anti-angiogenic soluble FLT1 (sFLT1). *LINC00607* CRISPR-knockout elevates sFLT1 and impairs angiogenesis, which is rescued by sFLT1 inhibition. *LINC00607* associates with U2 small nuclear RNA/BRG1 to modulate splicing. Junction-targeting splice-blocking morpholinos/antisense oligonucleotides selectively suppress sFLT1 and enhance sprouting. Reduced *LINC00607* in preeclampsia correlates with pathogenic sFLT1 elevation.

## Introduction

The vascular endothelium forms the barrier between the vessel wall and the blood. The endothelium plays a crucial role in regulating vascular permeability and tone, leukocyte and platelet adhesion, and tissue nutrient supply. The execution of these roles is regulated by aspects of the local environment, including cytokines, metabolites, shear stress, and cell-cell interactions.^1^ In response to hypoxia or local accumulation of angiogenic growth factors, endothelial cells sprout from preexisting vessels to form new vascular networks in a process termed angiogenesis. Angiogenesis is essential during development and plays a key role in wound healing and tissue adaptation. Defective or pathophysiological angiogenesis therefore contributes to clinical conditions such as tumor growth or proliferative vascular diseases of the eye.^2^^,^^3^

Alternative splicing (AS) is an essential step of RNA maturation that facilitates RNA and protein diversity from a single gene, enabling dynamic transcriptional adaptation to changes in the microenvironment.^4^^,^^5^ Splicing is crucial for cellular homeostasis, as aberrant AS contributes to the pathogenesis of numerous diseases.^6^^,^^7^^,^^8^ Various types of AS such as exon skipping, intron retention, mutually exclusive exons, and alternative 5′ and 3′ splice sites have been reported. All of these can result in the generation of multiple distinct products from the same gene.^6^ AS is regulated by splicing factors such as heterogeneous nuclear ribonucleoproteins and serine/arginine-rich regulators, which bind to *cis*-regulatory RNA elements within the nascent precursor RNA.^4^ AS is highly clinically relevant, and there are numerous diseases caused by aberrant splicing.^8^ Conversely, modulators of splicing, such as phosphorodiamidate morpholino (MO) oligomers, are being optimized to treat important diseases such as Duchenne muscular dystrophy.^9^

In endothelial cells, an important gene whose pre-mRNA undergoes AS is the vascular endothelial growth factor receptor 1 (*VEGFR1*, *FLT1*).^10^ Unlike VEGF, whose primary angiogenic function is mediated through VEGFR2 (KDR), FLT1 operates through multiple different isoforms with divergent functions. Among them are the membrane-bound canonical isoform FLT1 and a shorter, soluble isoform (sFLT1).^11^^,^^12^^,^^13^ FLT1 is important for angiogenic signaling through VEGF, particularly in response to hypoxia.^14^^,^^15^ By acting on the barrier between maternal and fetal circulation, sFLT1 prevents vascular overgrowth in the placenta.^16^ Overexpression of sFLT1 is associated with the development of preeclampsia (PE), a multisystem pregnancy disorder involving hypertension and proteinuria. Preeclampsia is one of the most prevalent and severe pregnancy-related diseases. It affects about 3%–5% of all pregnancies, is estimated to cause at least 42,000 maternal deaths annually, and is one of the main causes of prematurity.^17^^,^^18^^,^^19^ sFLT1 serum concentrations correlate with the severity of PE,^20^ and therefore sFLT1 in the maternal serum is used as a biomarker for the prediction and diagnosis of PE.^18^^,^^19^

Recent studies support a role of long non-coding RNAs (lncRNAs) in the control of splicing.^21^^,^^22^ lncRNAs are defined as RNA molecules longer than 200 nt that lack protein-coding potential.^23^^,^^24^ By acting on DNA, proteins, and other RNA molecules, lncRNAs can impact numerous cellular processes, including differentiation, migration, and gene expression.^24^ Numerous lncRNAs have been studied with respect to their functional relevance in endothelial cells.^25^ Among them are metastasis-associated lung adenocarcinoma transcript 1 (*MALAT1*)^26^ and SMARCA4 interacting SWI/SNF (switch/sucrose non-fermentable) chromatin remodeling complex scaffold lncRNA (*SMANTIS*).^27^^,^^28^ The role of lncRNAs in the endothelial control of splicing, however, remains fairly unexplored. So far, three different mechanisms have been proposed for lncRNA-mediated regulation of AS: (1) formation of RNA-RNA duplexes with precursor-RNA molecules, (2) interaction with specific splicing factors, and (3) influence on chromatin remodeling.^22^ In the complex regulation of AS, lncRNAs can directly impact their downstream targets and thereby promote disease progression.^29^ Mechanistic examples are *MALAT1*, which regulates the sub-nuclear localization and phosphorylation status of serine- and arginine-rich splicing factor 1 (SRSF1) by interacting with the SRSF protein kinase 1 or the protein phosphatases PP1/2A,^30^ and lncRNA *asFGFR2* (antisense fibroblast growth factor receptor 2), which modulates AS of *FGFR2* by enabling a splicing-specific chromatin signature that allows *FGFR2* exon IIIb inclusion.^31^

Previously, we and others reported that lncRNA *LINC00607* is highly expressed in endothelial cells and required for endothelial cell specification.^32^^,^^33^^,^^34^ *LINC00607* maintains the angiogenic capacity of human endothelial cells by facilitating ETS-related gene (ERG)-mediated gene transcription through an interaction with the chromatin remodeling protein BRG1.^34^ Given the role of *LINC00607* in transcriptional regulation and endothelial phenotypic control, we hypothesized that it governs AS programs critical for vascular function. Indeed, depleting *LINC00607* in endothelial cells resulted in AS of *FLT1*.

## Results

### Loss of *LINC00607* promotes the formation of soluble FLT1 through AS

**Figure 1:**
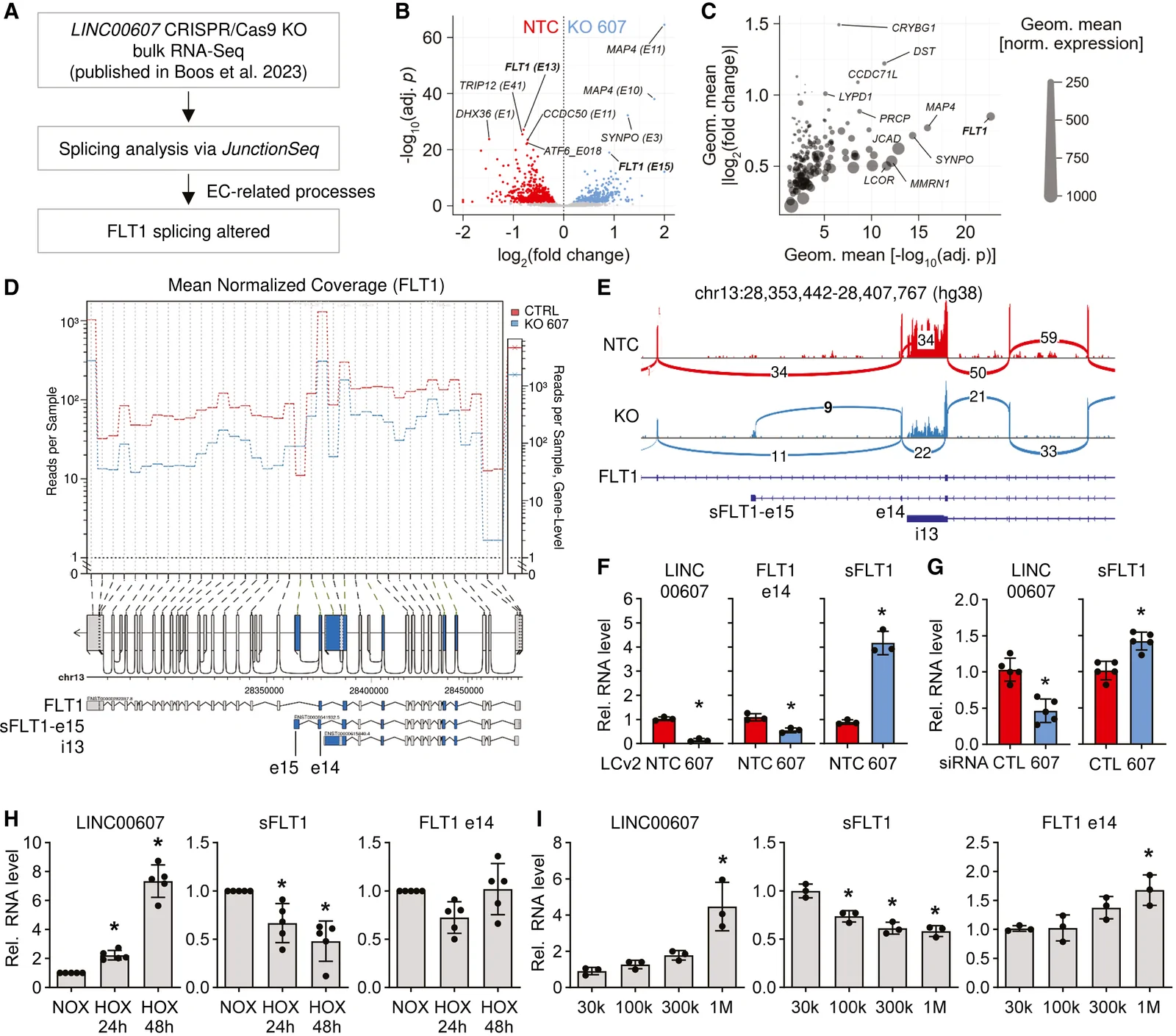
Knockout of *LINC00607* leads to the formation of soluble FLT1 (A) Scheme of the experimental workflow to reveal alternatively spliced targets in endothelial *LINC00607* knockout (KO) cells. (B) Volcano plot of differentially expressed exons (log_2_ fold expression changes) originating from the same gene of NTC versus *LINC00607* KO HUVECs. (C) Transcript isoform switches measured by mean log_2_ fold expression changes including only exons from the same gene with regulation in opposite directions. (D) Gene plot profile of *FLT1* from *JunctionSeq* analysis. Upper central plotting area displays mean normalized read counts of each *FLT1* exon in *LINC00607* KO (blue) and non-target control (NTC gRNA CTRL, red) and the gene-level normalized read counts on the right. Displayed below is the genomic locus of *FLT1*, its exon-intron structure, and a representation of *FLT1* isoforms. Indicated in blue are exons and introns overrepresented in *LINC00607* KO reads. (E) Sashimi plot of connecting reads of the RNA-seq indicating *FLT1* exons 11–15 in control (NTC) and *LINC00607* KO (KO). Red, NTC; blue, *LINC00607* KO. The numbers indicate the number of connecting reads. (F) Relative RNA level of *LINC00607* (left), *FLT1* exon 14 (center), and *FLT1* exon 14/15 (*sFLT1*) (right) by RT-qPCR after lentiCRISPRv2 (LCv2)-mediated KO of LINC00607. *n* = 3; paired *t* test. (G) Relative RNA level of *LINC00607* (left) and *FLT1* exon 14/15 (*sFLT1*) (right) by RT-qPCR after siRNA-mediated knockdown of *LINC00607*. Scrambled siRNA served as negative control (CTL). *n* = 5; paired *t* test. (H) RT-qPCR of *LINC00607*, *FLT1* exon 14–15 (*sFLT1*), and *FLT1* exon 14 in HUVECs treated with normoxia (NOX) or hypoxia (HOX) for 24 or 48 h. *n* = 5; one-way ANOVA with Dunnett post hoc test. (I) RT-qPCR of *LINC00607*, *FLT1* exon 14–15 (*sFLT1*), and *FLT1* exon 14 in different cell numbers of HUVECs on the same dish size (6-well) indicating weak to high confluency. *n* = 3; one-way ANOVA with Dunnett post hoc test. Error bars are defined as mean ± SD. ∗*p* < 0.05.

AS is a major source of gene product diversity and was initially thought to be regulated exclusively by proteins, splicing enhancers, and silencers.^4^^,^^5^^,^^6^ Recent work has implicated non-coding RNAs in the regulation of AS.^21^ Given that the endothelial-enriched lncRNA *LINC00607* is critical for the homeostasis and gene expression of human umbilical vein endothelial cells (HUVECs),^34^ we utilized our previously published RNA sequencing (RNA-seq)^34^ data to perform *JunctionSeq* analysis^35^ comparing the abundance of differentially spliced transcripts after lentiCRISPR-mediated *LINC00607* knockout (KO) (Figures 1A and S1A; Table S1). The analysis revealed that *LINC00607* KO results in the differential usage of more than 1,221 exons (Figure 1B), including also differentially expressed exons originating from the same gene, such as *FLT1*. Indeed, when considering likely transcript isoform switches, where two exons from the same gene were differentially expressed in opposite directions, *FLT1* exons 13 and 15 displayed the highest statistical significance of any exon pair in the analysis (Figure 1C), followed by microtubule-associated protein 4 (*MAP4*); no significant change of extended exon 3 of synaptopodin (*SYNPO*) could be observed (Figures S1B–S1H). Gene Ontology (GO) and Reactome enrichment analyses on the high-confidence isoform-switch candidates showed only mild but expected enrichment for tube and vasculature development and extracellular matrix organization (Tables 1 and 2).

**Table 1:** GO (GO biological process) analysis of *LINC00607* KO splicing candidates

GO biological process complete	Fold enrichment	Raw *p* value	FDR
Tube development (GO: 0035295)	4.2	2.10E−8	3.10E−4
Tube morphogenesis (GO: 0035239)	4.54	1.82E−7	1.34E−3
System development (GO: 0048731)	2.09	1.15E−6	2.42E−3
Blood vessel development (GO: 0001568)	4.88	1.06E−6	2.61E−3
Multicellular organism development (GO: 0007275)	2.02	9.40E−7	2.78E−3
Anatomical structure morphogenesis (GO: 0009653)	2.52	7.92E−7	2.92E−3
Regulation of cellular component biogenesis (GO: 0044087)	3.58	6.12E−7	3.01E−3
Vasculature development (GO: 0001944)	4.66	1.81E−6	3.34E−3
Angiogenesis (GO: 0001525)	5.85	2.95E−6	4.84E−3
Cell adhesion (GO: 0007155)	3.48	3.61E−6	5.33E−3
Blood vessel morphogenesis (GO: 0048514)	5.08	4.36E−6	5.85E−3
Anatomical structure development (GO: 0048856)	1.78	5.35E−6	6.58E−3
Developmental process (GO: 0032502)	1.71	6.45E−6	7.32E−3

The GO database released July 22, 2025 was used. Splice candidates with a log_2_ fold change >0.5 were considered for the analysis, yielding 108 candidate genes. FDR, false discovery rate; GO, Gene Ontology; KO, knockout.

**Table 2:** GO enrichment (Reactome pathways) analysis of *LINC00607* KO splicing candidates

Reactome pathways	Fold enrichment	Raw *p* value	FDR
ECM organization (R-HSA-1474244)	6.91	5.84E−7	1.53E−3
Integrin cell surface interactions (R-HSA-216083)	13.36	5.89E−6	7.71E−3
Laminin interactions (R-HSA-3000157)	24.95	1.90E−5	1.66E−2
ECM proteoglycans (R-HSA-3000178)	12.31	5.44E−5	3.56E−2

The GO database released July 22, 2025 was used. Splice candidates with a log_2_ fold change >0.5 were considered for the analysis, yielding 108 candidate genes. ECM, extracellular matrix; FDR, false discovery rate; GO, Gene Ontology; KO, knockout.

Because of the importance of FLT1 for VEGF signaling and endothelial cell function and the previously reported role of AS in the generation of functionally distinct FLT1 isoforms,^11^^,^^12^^,^^13^ we focused our analysis on this gene. RNA-seq and *JunctionSeq* data showed not only that *FLT1* is strongly downregulated (Figure 1D), as exemplified by the extended exon 13 (i13), but also that alternative exon 15 was upregulated, as indicated by more junction reads between exons 14 and 15 (Figure 1E).

This AS event generates the soluble isoform *sFLT1*, which is characterized by normal splicing of exons 13 and 14 but the inclusion of alternative exon 15. Alternative exon 15 acts as the terminal exon of *sFLT1* and is encoded in intron 14 of the canonical isoform. Alternative exon 15 contains a transcriptional stop and polyadenylation signal, and thus its inclusion leads to a truncated, non-membrane-bound short form of FLT1, which acts as a soluble scavenging receptor of VEGF.^36^ RT-qPCR with primers specific to the membrane-bound *FLT1* and the soluble-isoform *sFLT1* confirmed these findings: KO of *LINC00607* reduced the expression of exon 14 (common for both isoforms), while the KO increased exon 14/15 expression (specific for sFLT1) (Figure 1F).

Similarly, small interfering RNA (siRNA)-mediated knockdown of *LINC00607* corroborated the increase in *sFLT1* (Figure 1G). An inverse relationship between *LINC00607* and *sFLT1* was found in HUVECs exposed to 24 or 48 h of hypoxia, where *LINC00607* levels increased and *sFLT1* decreased (Figure 1H). Additionally, *sFLT1* levels declined with increasing confluence of endothelial cells, which mimics their quiescent state, while the expression of *LINC00607* was induced (Figure 1I). These data suggest that *LINC00607* suppresses the production of *sFLT1* and that loss of *LINC00607* may promote the formation of *sFLT1*.

### Reduced spheroid outgrowth by loss of *LINC00607* can be restored with antibodies against sFLT1

**Figure 2:**
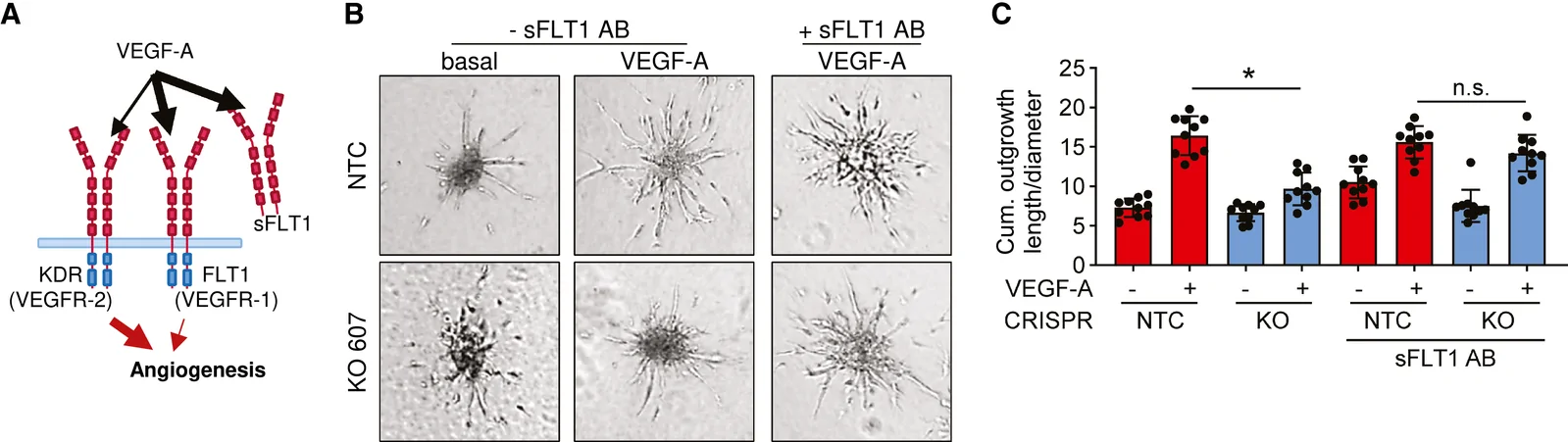
Spheroid outgrowth defects after KO of *LINC00607* can be blocked with an anti-sFLT1 antibody (A) Schematic representation of VEGF-A-mediated angiogenesis through FLT1 and KDR. (B) Spheroid outgrowth assay of HUVECs after CRISPR-Cas9-mediated *LINC00607* KO or NTC treated with or without sFLT1 neutralizing antibody (sFLT1 AB) is shown. Cells were also treated with or without VEGF-A (16 h, 30 ng/mL). (C) Quantification of the quotient of cumulative outgrowth length and respective spheroid diameter from spheroid outgrowth assay. *n* = 10; one-way ANOVA with Bonferroni post hoc test. Error bars are defined as mean ± SD. ∗*p* < 0.05; n.s., not significant.

Previously, we demonstrated that *LINC00607* maintains the angiogenic phenotype since its KO led to an inhibition of VEGF-mediated endothelial cell sprouting.^34^ Both VEGF receptors KDR and FLT1 are known to promote sprouting, whereas sFLT1 attenuates angiogenesis by limiting VEGF availability^16^ (Figure 2A). The involvement of *LINC00607* in *FLT1* transcription and splicing suggests that the pro-angiogenic capacity of *LINC00607* may be connected to *FLT1* isoform dynamics, evidenced by the increased generation of *sFLT1* following CRISPR-Cas9-mediated KO of *LINC00607*. To test this hypothesis, experiments were performed in the presence and absence of an sFLT1-blocking antibody . Indeed, KO of *LINC00607* attenuated VEGF-A-mediated endothelial sprouting, with normal angiogenic function being restored by the addition of an sFLT-1 blocking antibody (Figures 2B and 2C). This suggests that the increased formation of sFLT1 in *LINC00607* KO cells mediates the attenuated sprouting response.

### Exon 7 of *LINC00607* interacts with U2 snRNA in a BRG1-dependent manner

**Figure 3:**
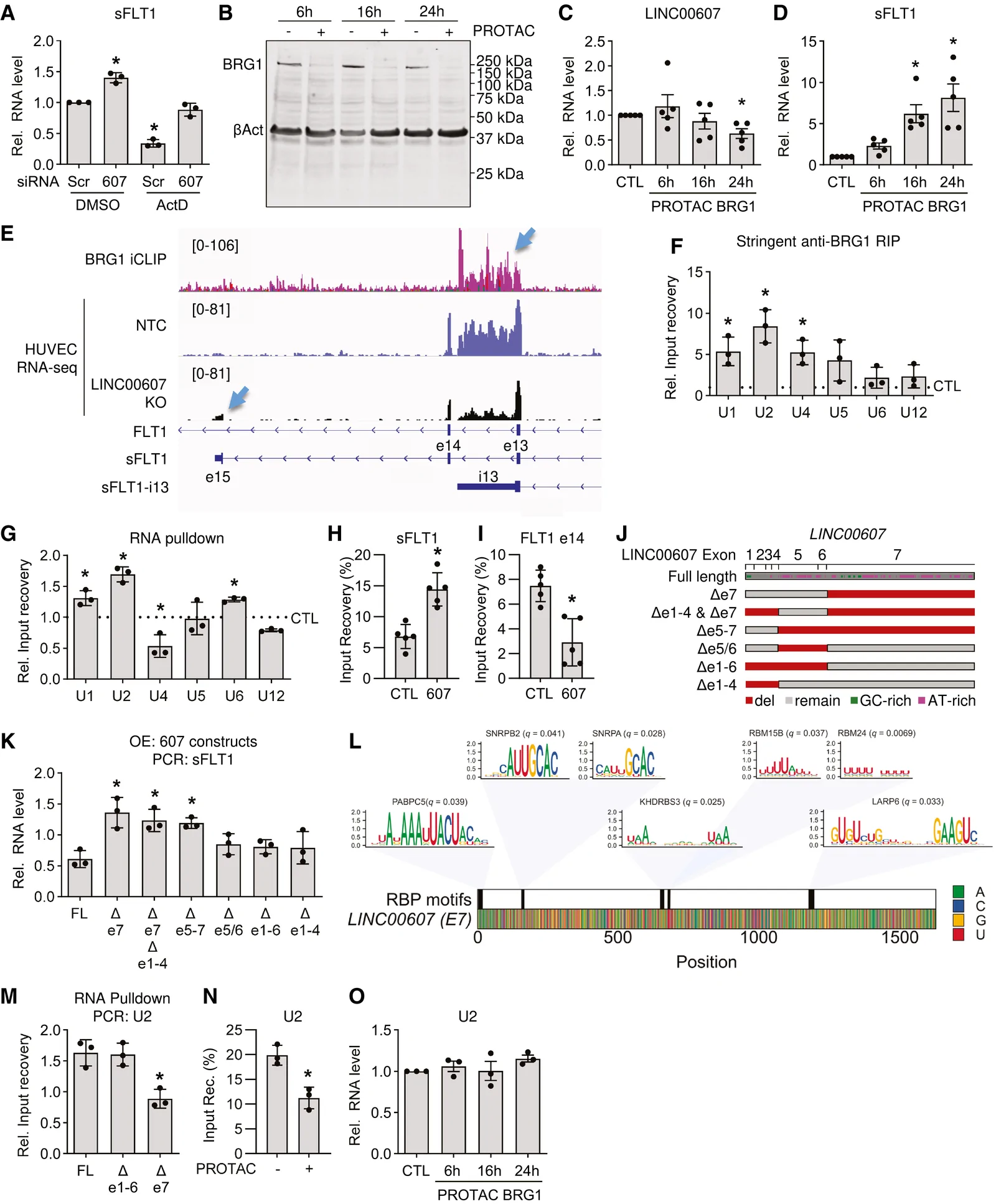
*LINC00607* exon 7 interacts with U2 snRNA in a BRG1-dependent manner (A) RT-qPCR after knockdown of *LINC00607* in HUVECs treated with or without actinomycin D (1 μM, 6 h). Expression levels of *sFLT1* were measured. *n* = 3; paired *t* test. (B) Western blot analysis of HUVECs treated with the BRG1 PROTAC AU-15330 (1 μM, 6–24 h). Anti-BRG1 is shown; anti-β-actin served as loading control. (C and D) RT-qPCR of *LINC00607* (C) or *sFLT1* (D) after treatment of HUVECs with the BRG1 PROTAC AU-15330 (1 μM, 6–24 h). *n* = 5; paired *t* test. (E) Integrated Genomics Viewer browser tracks (chr13: 28,365,319–28,395,906 [hg38]) of BRG1 iCLIP and RNA-seq of *LINC00607* KO and NTC datasets. The genomic locations of *FLT1* exon 15 (left, e15), exons 13 and 14 (center, e13, e14), as well as extended e13 (i13, belongs to sFLT-i13 isoform) are shown. The top arrow indicates BRG1 binding to *FLT1* RNA; the lower arrow indicates the appearance of reads in exon 15 after *LINC00607* KO. Numbers in brackets indicate number of reads. (F) Anti-BRG1 RNA IP with stringent washes, followed by RT-qPCR for individual U snRNAs. Relative input recovery of the respective U snRNA is shown. Immunoglobulin G (IgG) served as negative CTL and was set as 1. *n* = 3; one-way ANOVA with Bonferroni post hoc test. (G) RNA pulldown with *in vitro* transcribed and biotinylated *LINC00607*, followed by RT-qPCR for individual U snRNAs. Relative input recovery of the respective target is shown. IgG served as negative CTL and was set as 1. *n* = 3; one-way ANOVA with Bonferroni post hoc test. (H and I) RNA pulldown with *in vitro* transcribed and biotinylated *LINC00607* followed by RT-qPCR for *sFLT1* e14/15 (H) or *FLT1* e14 (I). Relative input recovery of the respective target is shown. IgG served as negative CTL. *n* = 5; paired *t* test. (J) Scheme of the individual *LINC00607* exonic (E) deletion mutants used. Red indicates deleted regions, gray indicates the remaining regions, green indicates the GC-rich regions, and purple indicates the AT-rich regions. (K) Relative RNA level of *FLT1* exon 14/15 (*sFLT1*) after overexpression of *LINC00607* full-length (FL) or the individual *LINC00607* deletion mutants. *n* = 3; samples were compared to FL using one-way ANOVA with Bonferroni post hoc test. (L) Scan of RNA binding protein motifs with the exon 7 sequence (E7) of *LINC00607* using the CISBP-RNA database. (M) RNA pulldown assay of *in vitro* transcribed and biotinylated *LINC00607* FL or deletion mutants. RT-qPCR for U2 snRNA is shown. *n* = 3; one-way ANOVA with Bonferroni post hoc test. (N) RNA pulldown assay of *in vitro* transcribed and biotinylated *LINC00607* FL after stimulation of cells with the BRG1 PROTAC for 16 h. RT-qPCR of U2 snRNA is shown. *n* = 3; paired *t* test. (O) RT-qPCR of U2 snRNA after treatment of HUVECs with the BRG1 PROTAC AU-15330 (1 μM, 6–24 h). *n* = 3; paired *t* test. Error bars are defined as mean ± SD. ∗*p* < 0.05.

To rule out the possibility that the accumulation of sFLT1 in the absence of *LINC00607* is due to heightened mRNA stability, we examined *sFLT1* exon 14/15 levels. Although *sFLT1* exon 14/15 expression levels increased in the absence of *LINC00607*, HUVECs treated with the transcription inhibitor actinomycin D displayed similar patterns of lower *sFLT1* exon 14/15 expression both in control and *LINC00607*-depleted conditions. This finding indicates that *LINC00607* does not affect *sFLT1* transcript stability (Figure 3A).

Previously, we demonstrated that *LINC00607* interacts with the SWI/SNF chromatin remodeling complex as seen by BRG1 pulldown in endothelial cells.^34^^,^^37^ To investigate whether the accumulation of *sFLT1* mRNA is a BRG1-dependent mechanism, we degraded BRG1 using the PROTAC AU-15330 (Figure 3B). BRG1 degradation resulted in decreased *LINC00607* expression and increased *sFLT1* levels (Figures 3C, 3D, and S2A). Interestingly, our previously published BRG1 individual-nucleotide resolution crosslinking and immunoprecipitation (iCLIP) data in HUVECs^37^ demonstrated that BRG1 interacts with intron 13 of the *FLT1* transcript, a region located near the *sFLT1*-relevant exon 15 (Figure 3E). iCLIP as well as RNA IP (± stringent wash buffers) of BRG1 followed by RT-qPCR indeed showed that BRG1 is able to interact with core U small nuclear RNAs (snRNAs) involved in splicing (Figures 3F and S2B; Table S2).

To test whether *LINC00607* also interacts with U snRNAs, *LINC00607* was transcribed *in vitro*, biotinylated, and subjected to RNA pulldown assays. Subsequent RT-qPCR analysis revealed that *LINC00607* interacts with U1, U2, and U6 snRNAs and *sFLT1* exon 14/15, but not with *FLT1* exon 14, U4, U5, or U12 snRNAs (Figures 3G–3I). To identify the essential regions of *LINC00607* required for its effect on sFLT1 exon 14/15, we generated mutants by removing specific exonic regions (Figure 3J). Overexpression of the full-length *LINC00607* transcript lowered *sFLT1* levels; however, this effect was abolished in mutants lacking exon 7 of *LINC00607* (Figure 3K). Scanning of the sequence of exon 7 for RNA binding protein motifs from the Catalog of Inferred Sequence Binding Preferences of RNA Binding Proteins (CISBP-RNA) database^38^ revealed that the exon harbors a potential binding motif for the protein SNRPB2 (*q* = 0.041), which lies in an accessible region (Figures 3L and S2C). SNRBP2 (alias U2B″) is known to bind the U2 snRNA hairpin IV,^39^ presenting a putative mechanism by which the association between *LINC00607* and U2 snRNA may take place. Additionally, the interaction of *LINC00607* with U2, but not with U1 or U6 snRNAs, was strongly reduced upon deletion of *LINC00607* exon 7 (Figures 3M, S3A, and S3B). This interaction was partially mediated by BRG1, as the PROTAC AU-15330 diminished the interaction of *LINC00607* with U2 without affecting the expression level of the U snRNAs (Figures 3N, 3O, and S3C–S3I). These data suggest that exon 7 of *LINC00607* interacts with U2 snRNA in a BRG1-dependent manner to regulate sFLT1 mRNA abundance.

### *LINC00607* and U2 snRNA interact with a specific sFLT1 splice-blocking MO

To address whether *LINC00607* is specifically involved in the regulation of *sFLT1* splicing, we developed a strategy involving splice-blocking MOs for binding the important intron 14/exon 15 splice site, which is central for the generation of sFLT1.

**Figure 4:**
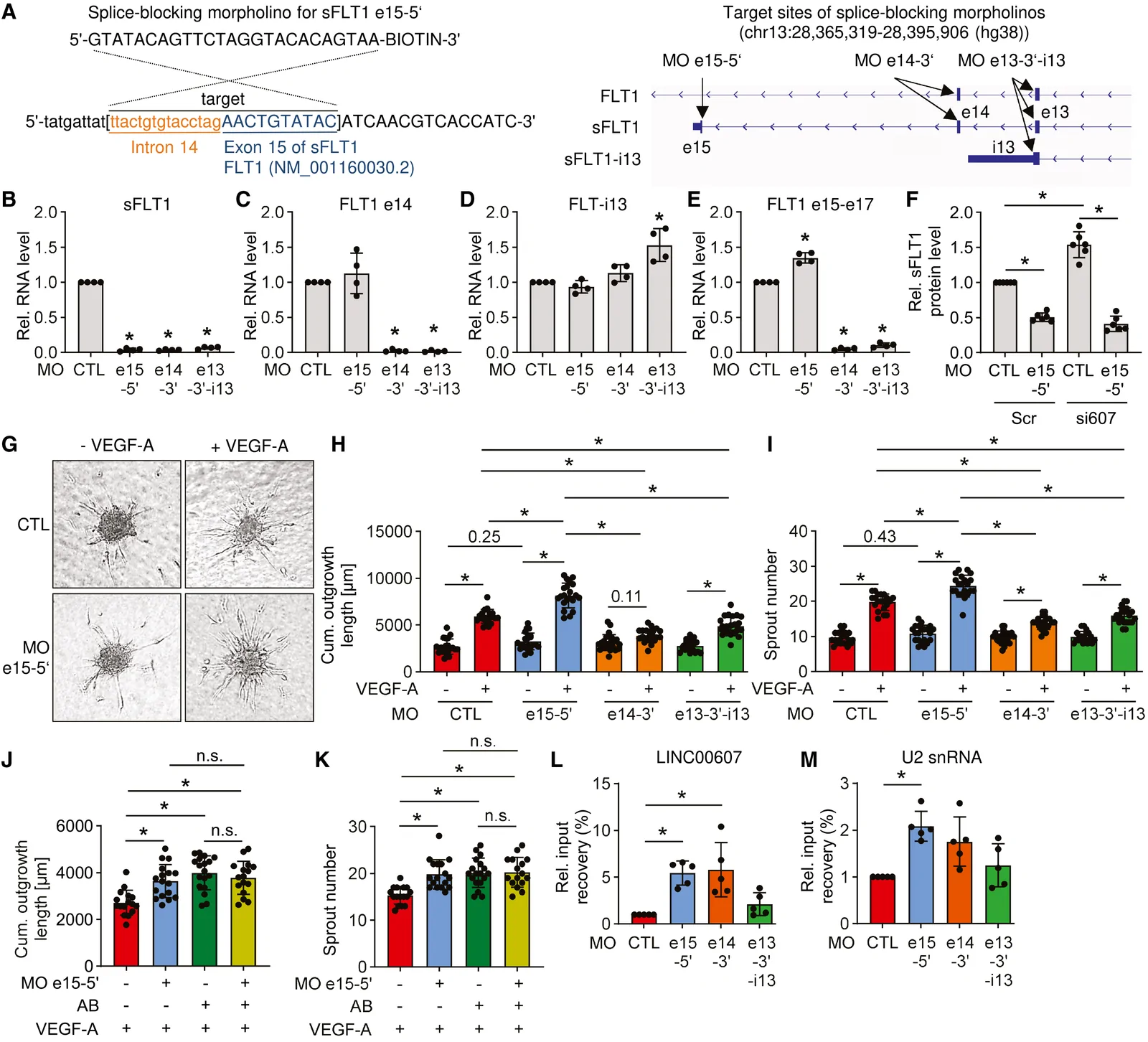
The specific sFLT1 e15-5′ splice-blocking morpholino abolishes sFLT1 production and interacts with *LINC00607* and U2 snRNA (A) Left: sequence of the splice-blocking morpholino (MO) e15-5′ and its binding to the intron 14/exon 15 junction of the *sFLT1* pre-mRNA. The splice-blocking MO is oriented reverse-complementary to the target sequence. The target sequence is shown in square brackets. Lowercase letters indicate the intronic sequence, whereas uppercase letters indicate the exonic sequence. Right: target sites of the splice-blocking MOs e15-5′ (here shown as 15I/10E), e14-3′, and e13-3′-i13 in the genomic context (chr13: 28,365,319–28,395,906 [hg38]). Arrows point to the intron/exon borders of *FLT1*. (B–E) RT-qPCR of *sFLT1* (B), *FLT1* exon 14 (e14) (C), *FLT1*-intron 13 (i13) (D), and *FLT1* exons 15–17 (e15–e17) (E) after electroporation of HUVECs with the indicated splice-blocking MOs (10 μM, 24 h). Data are normalized to *β-actin*. *n* = 4; paired *t* test. (F) sFLT1 ELISA after *LINC00607* knockdown (72 h) and/or treatment with MO e15-5′ (10 μM, 24 h). HUVEC medium was measured. *n* = 6; one-way ANOVA with Tukey post hoc test. (G) Selected images from the spheroid outgrowth assay of HUVECs electroporated with the MO e15-5′, followed by treatment with VEGF-A (30 ng/mL, 16 h). (H and I) Quantification of the spheroid outgrowth assay by cumulative sprout length (H) and number of sprouts (I). *n* = 21; one-way ANOVA with Tukey post hoc test. (J and K) Spheroid outgrowth assay using the sFLT1-lowering e15-5′ MO alone or combined with administration of the anti-sFLT1 antibody [AB] followed by treatment with VEGF-A (30 ng/mL, 16 h) in HUVECs. Quantification of the spheroid outgrowth assay by cumulative sprout length (J) and number of sprouts (K) is shown. One-way ANOVA with Tukey post hoc test. (L and M) RNA streptavidin pulldown assay of the indicated 3′ biotinylated splice-blocking MOs in HUVEC followed by RT-qPCR for *LINC00607* (L) and U2 snRNA (M). *n* = 5; one-way ANOVA with Dunnett post hoc test. n.s., not significant. Error bars are mean ± SD. ∗*p* < 0.05.

First, a 25-nt-long splice-blocking MO (named MO e15-5′) was designed, covering the intron 14/exon 15 splice site (15 nt bind to the intronic [I] site, 10 nt bind to the exonic [E] site) that is uniquely used to generate the sFLT1 isoform (Figure 4A). As additional controls to the standard negative control, splice-blocking MOs against other *FLT1* splice sites were designed, among them one covering exon 14/intron 14 (named MO e14-3′) and one covering exon 13/intron 13 (named MO e13-3′-i13). Interestingly, all splice-blocking MOs strongly decreased *sFLT1* abundance in HUVECs (Figure 4B), which was expected as targeting the upstream e14-3′ or the e13-3′ regions interferes with splice-site usage shared between the soluble and membrane-bound transcripts. MO e15-5′ acted specifically on *sFLT1*, but it did not reduce the abundance of *FLT1* exon 14, which is required for the membrane-bound FLT1 isoform (Figure 4C). Also, the abundance of *FLT1-i13* and the exons 15–17 from the canonical membrane-bound *FLT1* isoform were not decreased by MO e15-5’ (Figures 4D and 4E). In contrast, MO e14-3′ and MO e13-3′-i13 also reduced *FLT1* exon 14 and exon 15–17 abundance, but not *FLT1-i13* as MO e13-3′-i13 binds to an alternative splice site at the end of exon 13, which is not affected for *FLT1-i13* as this isoform transitions into intron 13 (i13) without splicing (Figures 4A and 4C–4E). None of the MOs changed the expression of *LINC00607* (Figure S4A). Furthermore, MO e15-5′ reduced in an enzyme-linked immunosorbent assay (ELISA) the protein abundance of sFLT1, even after *LINC00607* depletion (Figure 4F). These data demonstrate that all splice-blocking MOs target FLT1, and MO e15-5′ is the only efficient and specific sFLT1 blocker.

Second, we tested the MO e15-5′ for its effects on physiological function. Application of the MO e15-5′ did not increase apoptosis compared with the negative control MO in HUVECs (Figure S4B). A spheroid outgrowth assay of HUVEC transfected with the splice-blocking MOs indicated that MO e15-5′ was the only MO that increased sprouting (Figures 4G–4I), as it specifically induced a strong *sFLT1* reduction, leaving the pro-angiogenic membrane-bound *FLT1* RNA unchanged. Importantly, additional administration of the anti-sFLT1 AB had no additional effect on increasing sprouting after MO e15-5′ treatment. When administrated separately in individual experiments, each treatment increased sprouting, but together (sFLT1 AB + MO e15-5′) there was no additive effect (Figures 4J and 4K), demonstrating that both approaches converge on the same molecular principle.

We decided to further characterize the intron 14/exon 15 splice-blocking MO and modified the MO coverage and length; we also tested whether antisense oligonucleotides (ASOs) instead of MOs yield a similar response (Figure S4C). Interestingly, *sFLT1* abundance also strongly decreased by shortening the splice-blocking MO (from originally MO e15-5′ 15I/10E to 8I/7E) or changing its target site completely to the intronic site (25I/0E) without affecting *FLT1* exon 14 or *LINC00607* expression (Figures S4D–S4F). When using ASOs instead of MOs, we observed that these also decrease the abundance of *sFLT1*, although not as efficiently as splice-blocking MOs (Figures S4G and S4H). Importantly, this approach also increased VEGF-A mediated angiogenic sprouting, and the increasing effect was abolished when using a modified e15-5′ ASO, where the intron 14/exon 15 binding sequence was mutated (e15-5′ ΔAS, mutated from TTCT [AGAA on pre-mRNA] to GGGG) (Figures S4I and S4J). These results strongly suggest that targeting the intron 14/exon 15 splice site with MOs or ASOs is effective.

To show that *LINC00607* and U2 snRNA are specifically involved in *FLT1* splicing of alternative exon 15, a biotin tag was added at the 3′end of the MOs, and an RNA pulldown was performed to identify whether *LINC00607* and U2 snRNA specifically interact with these splice sites. *LINC00607* and U2 snRNA interacted with the splice site at e15-5′, and to a much weaker extent with the preceding splice site e14-3′, where U2 snRNA was tendentially associated to, but not with the splice site at exon 13-3′-i13 (Figures 4L and 4M). These experiments demonstrate that MO e15-5′ (1) binds to *LINC00607* and U2 snRNA, (2) is a specific sFLT1 blocker, (3) displays tolerability without increasing apoptosis, and (4) leads to increased VEGF-A-mediated angiogenic sprouting.

### *LINC00607* is critical for *sFLT1* production in trophoblasts and differentially expressed in PE

To explore potential genetic influences on this pattern of regulations, we performed expression quantitative trait loci (eQTL) analysis. We tested whether single-nucleotide polymorphisms (SNPs) near *FLT1* and *LINC00607* (±500 kb window around the gene regions) affect the transcription of sFLT1 (ENST00000541932), *FLT1* (ENST00000282397), and *LINC00607* (ENST00000445174) in aorta, coronary artery, tibial artery, adipose subcutaneous tissue, adipose visceral (omentum) tissue, heart-atrial appendage, heart-left ventricle, liver, spleen, skeletal muscle, and blood. Unfortunately, among the shared SNPs that regulate *LINC00607* and *FLT1* isoforms, all regulations keep the same directionality (Tables S3 and S4).

**Figure 5:**
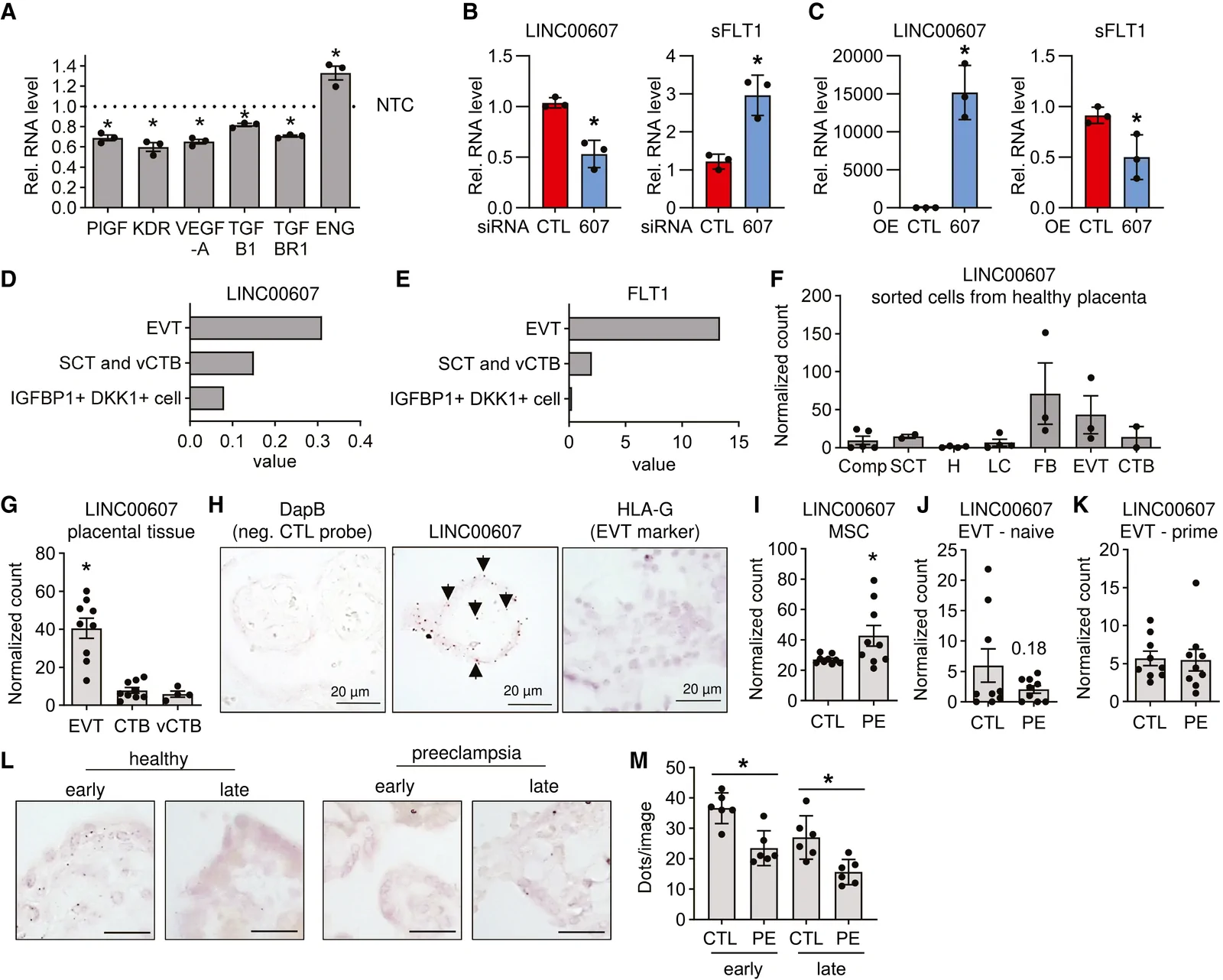
*LINC00607* is highly expressed in extravillous trophoblast cells in the placenta and reduced in preeclampsia sections from early- and late-stage pregnancy (A) Relative RNA level of *PlGF*, *KDR*, *VEGF-A*, *TGFB1*, *TGFBR1*, and *ENG* by RNA-seq after lentiCRISPRv2-(LCv2)-mediated KO of *LINC00607*. *n* = 3; paired *t* test. (B) Relative RNA level of *LINC00607* (left) and *FLT1* exon 14/15 (*sFLT1*) (right) by RT-qPCR after siRNA-mediated knockdown of *LINC00607* in JEG-3 trophoblasts for 48 h. Scrambled siRNA served as negative CTL. *n* = 3; paired *t* test. (C) Relative RNA level of *LINC00607* (left) and *FLT1* exon 14/15 (*sFLT1*) (right) by RT-qPCR after overexpression (OE) of pcDNA3.1+LINC00607 FL in JEG-3 trophoblasts for 24 h. An empty pcDNA3.1+ served as negative CTL. *n* = 3; paired *t* test. (D and E) *LINC00607* and *FLT1* expression in placental cell types from publicly available scRNAseq dataset (Vento-Tormo et al.^40^). (F) *LINC0607* expression in sorted cells from healthy placenta. Data originated from GSE182381. Comp, composite; CTB, cytotrophoblast; EVT, extravillous trophoblast; FB, fibroblast; H, Hofbauer cells; LC, leukocyte; SCT, syncytiotrophoblast. (G) *LINC00607* expression levels in placental tissue. Data originated from GSE256412. One-way ANOVA with Dunnett T3 post hoc test. vCTB, villous cytotrophoblasts. (H) RNAscope of *LINC00607* RNA in healthy term placenta sections. Negative CTL probe: DapB. EVT marker: *HLA-G*. 50×, hematoxylin. (I–K) *LINC00607* expression levels in MSCs (I), naive EVT (J), and prime EVT (K). Data originated from GSE243579. *n* = 9; unpaired *t* test. MSC, mesenchymal stem cell. (L) RNAscope of *LINC00607* in healthy and preeclamptic placental sections of early- and late-stage pregnancy. Scale bars, 20 μm. (M) Quantification of RNAscope of *LINC00607* shown in (L). CTL, healthy; PE, preeclampsia. *n* = 6; one-way ANOVA with Tukey post hoc test. Error bars are defined as mean ± SD. ∗*p* < 0.05.

An increased expression of sFLT1 in combination with reduced VEGF signaling and lower placental growth factor (PlGF) levels are characteristic for PE.^17^^,^^19^ In HUVEC, KO of *LINC00607* reduced the expression of *PlGF*, *KDR*, *VEGF-A*, *TGFB1*, *TGFBR1*, and increased the expression of *ENG* (Figure 5A). As these expression differences are described to be present in PE,^41^ we expanded our functional data to the trophoblast cell model JEG-3. Depletion of *LINC00607* with siRNAs increased *sFLT1* expression levels (Figure 5B), whereas plasmid-based overexpression of *LINC00607* decreased *sFLT1* expression levels (Figure 5C). These results indicate that *LINC00607* is also important for the regulation of *sFLT1* in JEG-3 trophoblasts. To determine whether these findings are reflected in the human placenta, publicly available human placental RNA-seq datasets were analyzed. Data from the cell atlas of fetal gene expression^42^ demonstrate that both *LINC00607* and *FLT1* are expressed in fetal placental extravillous trophoblasts (EVTs), syncytiotrophoblasts, and villous cytotrophoblasts (vCTBs) (Figures 5D and 5E). In addition, two publicly available datasets—one profiling sorted cells isolated from healthy placentas^43^ and another with EVTs, CTBs, and vCTBs^44^—confirmed that *LINC00607* expression is highest in EVTs (Figures 5F and 5G).

Additionally, RNA *in situ* hybridization (RNAscope) performed on human term placental samples demonstrated that *LINC00607* is indeed expressed in placental tissue (Figure 5H). A cohort study of patients with and without PE, in which pluripotent stem cell-based modeling of PE was performed, included reprogrammed naive and prime EVTs.^45^ Re-analysis of the data revealed that *LINC00607* was non-significantly reduced during PE in naive EVTs. Of note, this effect was not observed in mesenchymal stem cells (MSCs) or prime EVTs (Figures 5I–5K).

To further investigate the potential differential expression of *LINC00607* in PE, RNAscope was performed on placental sections from early- and late-stage pregnancies from healthy and PE patients. This revealed that *LINC00607* was downregulated in early and late-stage PE compared with healthy pregnancies, suggesting that the decrease in *LINC00607* in EVTs during PE may contribute to the upregulation of sFLT1 (Figures 5L and 5M).

Collectively, these findings suggest that *LINC00607* may play a role in the pathogenesis of PE, potentially through its involvement in sFLT1 AS.

## Discussion

Pathological angiogenesis contributes to a variety of disease conditions, among them cancer and cardiovascular diseases^2^^,^^3^ including the pregnancy-related disorder PE.^17^ The findings presented here establish *LINC00607* as a critical regulator of sFLT1 production through AS. Loss of *LINC00607* leads to significant splicing alterations, particularly affecting *FLT1*, where the inclusion of alternative exon 15 promotes the production of *sFLT1*. This soluble isoform sequesters VEGF, effectively attenuating VEGF receptor-mediated signaling and impairing angiogenesis. The generation of a specific splice-blocking MO (e15-5′, with its coverage 15I/10 E), to which *LINC00607* and U2 snRNA bound, completely abolished sFLT1 abundance and increased VEGF-A-mediated angiogenesis. *LINC00607* may therefore function as a modulator between pro- and anti-angiogenic VEGF-dependent signaling.

AS has long been recognized as a key contributor to transcriptome and proteome diversity. Although splicing itself requires several ncRNAs, AS is generally attributed to protein-mediated mechanisms. For example, the AS-dependent formation of *sFLT1* in HUVECs has been reported to be a consequence of the attenuated binding of JMJD6 to U2AF65^46^ and correlated to the expression of neuro-oncological ventral antigen 2 (*NOVA2*), although no significant regulation of sFLT1 by NOVA2 was found in the placenta.^47^ The present work supports the emerging role of lncRNAs in AS. *JunctionSeq* and RNA-seq analyses revealed that *LINC00607* KO induced widespread splicing changes, with exon 15 inclusion in *FLT1* as a key event. This splicing pattern results in the upregulation of sFLT1, while in parallel the membrane-bound *FLT1* isoform is downregulated, collectively shifting the vascular balance toward an anti-angiogenic situation. Functional assays confirmed the biological relevance of these splicing events. The reduction in VEGF-mediated endothelial sprouting caused by *LINC00607* KO was prevented by an sFLT1-blocking antibody.

Mechanistically, the present work suggests that an interplay between *LINC00607*, the chromatin remodeler BRG1, and U2 snRNA might contribute to the AS events. It was previously shown that BRG1 interacts with snRNPs in HeLa cells using IP experiments involving the core snRNP Ab Y12,^48^ while a screen in HeLa cells revealed an interaction of BRG1 with U2-B″.^49^ Interestingly, in the cervical carcinoma cell line C33A, which is deficient in SWI/SNF, an exogenous overexpression of BRG1 affected exon inclusion and skipping of exons.^50^ The study also showed that BRG1 might facilitate the binding of RNA binding factors to chromatin and RNA at alternative exons.^50^ In the present study, rapid degradation of BRG1 by a PROTAC approach increased *sFLT1* levels, supporting its role as a repressor of *FLT1* alternative exon inclusion and a mediator of *LINC00607* regulatory functions. *LINC00607* interacted with U2 snRNA through exon 7. The analysis of the different mutants also indicated that AT- or GC-rich regions of *LINC00607* are not important for this interaction. This behavior positions *LINC00607* as a potential scaffold for *FLT1* splicing regulation, linking chromatin state and transcript processing in endothelial cells.

Given the important role of *LINC000607* in AS and in endothelial phenotype control,^34^ it is tempting to speculate about the disease relevance of *LINC00607*. *LINC00607* expression was non-significantly downregulated during PE in naive EVTs. Notably, the inverse correlation between *LINC00607* and *sFLT1* levels in hypoxic endothelial cells and the reduction of *LINC00607* in preeclampsic placental tissues and in JEG-3 trophoblasts support the hypothesis that *LINC00607* negatively regulates *sFLT1* production.

PE, a severe multisystem pregnancy disorder often induced by poor placentation, is characterized by sFLT1 production.^17^^,^^19^ The present findings motivate further exploration into their relationship with *LINC00607*. Increased sFLT1 production is accompanied by a lower yield of the pro-angiogenic PlGF,^19^ which was also observed in the present study. Similar to our observation in endothelial cells, the reduction of *LINC00607* expression in RNAscope images of PE patient material would match the present literature with elevated sFLT1 levels in EVTs of PE patients. This mechanism would be remarkable for two reasons. First, it would suggest that the regulation of the AS of *FLT1* by *LINC00607* is not unique to endothelial cells but rather a general phenomenon. Second, and potentially even more important, it would suggest a functional importance of *LINC00607* in trophoblasts, which is an unexpected finding. Our experiments in JEG-3 trophoblasts support that notion. In fact, we have previously reported that *LINC00607* is highly endothelial enriched.^34^ The data of the present study not only implies a similarity of endothelial cells and trophoblasts with respect to their transcription factor profile but they also suggest that the primary site of action of *LINC00607* could be the placenta. In fact, many lncRNAs exhibit cell-type-specific expression patterns,^24^ and some lncRNAs are only transiently expressed or functional relevant during embryonic development. Potentially, *LINC00607* is one of these lncRNAs.

The data of the present study also advocate *LINC00607* as a potential biomarker or even therapeutic target for PE. A critical next step is to validate the *LINC00607*-*sFLT1* axis in a new, prospectively collected cohort of PE placentas using techniques capable of direct sFLT1 protein quantification (e.g., ELISA) or isoform-specific transcript analysis. Given that up until now the treatment options for PE were very limited and that preterm delivery is often the only cure, novel therapeutic avenues for the treatment of PE are acutely required.^19^ Therefore, our splice-blocking MOs and ASOs are a novel approach. We demonstrate that targeting the intron 14/exon 15 splice site with these specific MOs can lead to a more than 95% reduction in *sFLT1* abundance by increasing VEGF-A-mediated spheroid outgrowth. The targeting site we tested here also with ASOs was flexible in a range of up to 50 nt, having 25 nt inside the intron 14 and 25 nt inside the alternative exon 15. Moreover, the splice-blocking MO e15-5′ (with its configuration 15I/10E) was tolerated by HUVECs without increasing apoptosis and supported the mechanism of protection of *sFLT1* alternative exon 15 inclusion by *LINC00607*.

Direct targeting of sFLT1 is an approach that has already been explored. In animal models of PE, siRNAs against *sFLT1* reduced the severity of the disease.^51^ Recently, lipid nanoparticles (LNPs) were used to treat hypoxia- or inflammation-induced PE in murines. The LNP encapsulating VEGF mRNA resolved maternal hypertension until the end of gestation, improved fetal health, and partially restored placental vasculature.^52^ Importantly, recent delivery advances further strengthen the translational feasibility of ASO-based strategies, including context-responsive ionizable LNPs for lncRNA therapy and ligand/aptamer-functionalized LNPs enabling cell-selective nucleic acid delivery *in vivo*.^53^^,^^54^ Moreover, self-transfecting guanidinium-linked MO-phosphorodiamidate MO oligonucleotide chimera approaches illustrated that inhibition of a target gene could be possible without using any delivery vehicle.^55^ Although these data are interesting, it should be noted that PE is a disease found only in humans.^56^ This is remarkable, as *LINC00607* expression is restricted to primates, and the lncRNA is not conserved in other lower mammals. Therefore, a limitation of this study is that we were not able to perform *in vivo* validation, which is not only because of the primate restriction of *LINC00607* but also due to the lack of a suitable rodent model for the human *sFLT1* splice event.

Our study identifies *LINC00607* as a key regulator of *FLT1* AS and angiogenesis, with significant implications for endothelial biology and disease. Future studies involving the splice-blocking MOs should focus on elucidating the precise molecular mechanisms of *LINC00607*-mediated splicing regulation and its therapeutic potential in vascular pathologies.

## Materials and methods

### Materials

The following chemicals and concentrations were used for stimulation: human recombinant VEGF-A 165 (R&D Systems, catalog no. 293-VE), PROTAC AU-15330 (MedChemExpress, catalog no. HY-145388, 1 μM used) and actinomycin D (Sigma, catalog no. A9415). The following Abs were used: anti-β-actin (Sigma-Aldrich, catalog no. A1978), VEGF receptor 1 (soluble) polyclonal Ab (Thermo Fisher Scientific, catalog no. 36-1100), anti-BRG1 (Abcam, catalog no. ab110641, for RNA IP experiments), and anti-BRG1 (Bethyl Laboratories, catalog no. A303-877 A, for western blot).

### Cell culture

Pooled HUVECs were purchased from PromoCell (catalog no. C-12203, lot nos. 405Z013, 408Z014, and 416Z042). The HUVEC batches originated from the umbilical cord/umbilical vein of White individuals (405Z013: two males, one female; 408Z014: two males, one female; 416Z042: two males, two females). HUVECs were cultured in a humidified atmosphere with 5% CO_2_ at 37°C. Gelatin-coated dishes (Corning, catalog no. 356009) were used to culture the cells. Endothelial growth medium kit enhanced (EGM) (PeloBiotech, catalog no. PB-C-MH-100-2199) without hydrocortisone supplemented with growth factors (EGF, basic FGF, insulin growth factor, VEGF, heparin, and l-glutamine), 8% fetal calf serum (FCS) (Biochrom, catalog no. S0113), penicillin (50 U/mL), and streptomycin (50 μg/mL) (Gibco/Life Technologies, catalog no. 15140-122) was used to culture HUVEC. For each experiment, at least three different batches of HUVECs from passage three were used. For hypoxia experiments, HUVECs were incubated for 24 or 48 h in a SciTive Workstation (Baker Ruskinn) at 1% O_2_ and 5% CO_2_.

JEG-3 trophoblasts were purchased from American Type Culture Collection and cultured in a humidified atmosphere of 5% CO_2_ at 37°C in MEM (Gibco, catalog no. 31095-029) supplemented with 1% non-essential amino acids (Sigma-Aldrich, catalog no. M7145), 1 mM sodium pyruvate (Sigma-Aldrich, catalog no. S8636), 8% FCS (Biochrom, catalog no. S0113), and gentamycin (Thermo Fisher Scientific, catalog no. 15750-045).

### RNA isolation, reverse transcription, and RT-qPCR

Total RNA isolation was performed with the RNA Mini Kit (Bio&Sell) according to the manufacturer’s protocol. Reverse transcription was performed with SuperScript III Reverse Transcriptase (Thermo Fisher) using oligo(dT)23 together with random hexamer primers (Sigma). RT-qPCR was done using ITaq Universal SYBR Green Supermix and ROX as reference dye (Bio-Rad, catalog no. 1725125) in an AriaMX cycler (Agilent). Relative expression of target genes was normalized to β-actin. Expression levels were analyzed by the delta-delta Ct method with the AriaMX qPCR software (Agilent Aria 1.7). The following oligonucleotides were used for amplification: β-actin, forward 5′-AAA GAC CTG TAC GCC AAC AC-3′ and reverse 5′-GTC ATA CTC CTG CTT GCT GAT-3′; *LINC00607*, forward 5′-GGA TGC AGC AGA AGA GGA TG-3′ and reverse 5′-GAC AGG ACT GGC AGT AAT CG-3′; U1 snRNA, forward 5′-TGA TCA CGA AGG TGG TTT TCC-3′ and reverse 5′-GCA CAT CCG GAG TGC AAT G-3′; U2 snRNA, forward 5′-TTC TCG GCC TTT TGG CTA AG-3′ and reverse 5′-CTC CCT GCT CCA AAA ATC CA-3′; U4 snRNA, forward 5′-GCC AAT GAG GTT TAT CCG AGG-3′ and reverse 5′-TCA AAA ATT GCC AAT GCC G-3′; U5 snRNA, forward 5′-TGG TTT CTC TTC AGA TCG CAT AAA-3′ and reverse 5′-CCA AGG CAA GGC TCA AAA AAT-3′; U6 snRNA, forward 5′-CTC GCT TCG GCA GCA CA-3′ and reverse 5′-AAC GCT TCA CGA ATT TGC GT-3′; U12 snRNA, forward 5′-GCC CGA ATC CTC ACT GCT AA-3′ and reverse 5′-TCG CAA CTC CCA GGC ATC-3′; FLT1, 5′-AGG TTT CGC AGG AGG TAT GG-3′ (only for exon 14) and 5′-CAG CAG TTC CAC CAC TTT AG-3′ (for exon 14 and for sFLT1) and 5′-TGA CGA TGG TGA CGT TGA TG-3′ (only sFLT1); FLT1-i13, 5′-GAG GGT GGG AAA GAC ATT AG-3′ and 5′-CAG TCA GTC CTG CTG ATG TG-3′; FLT1 e15–e17, 5′-AAC CAG AAG GGC TCT GTG GAA AGT-3′ and 5′-CAA ACT CCC ACT TGC TGG CAT CAT-3′; MAP4 exon 10/11, forward 5′-CAA AGC AAC CAG GCA CTA AG-3′ and reverse 5′-CTG TGC TAA CAC CAG AAG TC-3′; MAP4 exon 22, forward 5′-CAG TGC CTT GTG GAG GTT TG-3′ and reverse 5′-TGG TCT CTA CGG AGG AAA GC-3′; SYNPO last exon end, forward 5′-GTG AGG GAA GGG TTG GAA TG-3′ and reverse 5′-GAG GCA ATC CTG AGC CTA AG-3′; SYNPO last exon begin, forward 5′-ACC CAC CGG CAA CTG TTG TC-3′ and reverse 5′-AGC AGG CCC TGT GCC ATT AG-3′.

### siRNA-mediated knockdown

For siRNA treatments, HUVECs or JEG-3 cells (80%–90% confluent) were transfected with Lipofectamin RNAiMAX transfection reagent according to the instructions provided by Thermo Fisher Scientific. The following Silencer Select siRNAs were used: siLINC00607 (Thermo Fisher Scientific, catalog no. s56344) and Silencer Select Negative Control No. 1 siRNA (Thermo Fisher Scientific, catalog no. 4390844). All siRNA experiments were performed for 48 h.

### LentiCRISPRv2

Guide RNAs (gRNAs) targeting *LINC00607* were designed with CRISPOR (http://crispor.tefor.net/).^57^ A dual gRNA approach was used to facilitate the KO of *LINC00607*, gRNA1 targeted a region downstream of the transcription start site (TSS), and the gRNA2 targeted a region upstream of the TSS of *LINC00607*. The gRNAs were cloned into the lentiCRISPRv2 vector backbone with Esp3I (Thermo Fisher, catalog no. FD0454) according to the standard protocol.^58^ LentiCRISPRv2 was a gift from Feng Zhang (Addgene, plasmid no. 52961). The following oligonucleotides were used for cloning: gRNA1, 5′-CAC CGC ATG TGC CCC CTT TGT TGA A-3′ and 5′-AAA CTT CAA CAA AGG GGG CAC ATG C-3′; and gRNA2, 5′-CAC CGC AGT GTG TCA TGT TAT CTT G-3′ and 5′-AAA CCA AGA TAA CAT GAC ACA CTG C-3′. After cloning, the gRNA-containing LentiCRISPRv2 vectors were verified by Sanger sequencing with hU6-F. Lentivirus was produced in Lenti-X 293 T cells (Takara Bio, catalog no. 632180,) using polyethylenamine (Sigma-Aldrich, catalog no. 408727), psPAX2, and pVSVG (pMD2.G). psPAX2 and pMD2.G were a gift from Didier Trono (Addgene, plasmids 12260 and 12259). LentiCRISPRv2-produced virus was transduced in HUVECs (p1) with polybrene transfection reagent (MerckMillipore, catalog no. TR-1003-G), and antibiotic selection was performed for 6 days prior to RNA and genomic DNA isolation.

### Spheroid outgrowth assay

Spheroid outgrowth assays in HUVECs were performed as previously described.^59^ Spheroids were stimulated with the indicated concentrations of VEGF-A 165 for 16 h. Images were captured using an Axiovert 135 microscope (Zeiss). Cumulative sprout lengths and spheroid diameters were quantified using AxioVision software (Zeiss).

### Nuclear protein isolation and western analysis

For nuclear protein isolation, cells were resuspended in hypotonic buffer (10 mM HEPES pH 7.6, 10 mM KCl, 0.1 mM EDTA pH 8.0, 0.1 mM EGTA pH 8.0, 1 mM DTT, and 40 μg/mL PMSF) and incubated on ice for 15 min. Nonidet was added to a final concentration of 0.75%, and cells were centrifuged (1 min, 4°C, 16,000 × *g*). The nuclear pellet was washed twice in hypotonic buffer, lysed in high salt buffer (20 mM HEPES pH 7.6, 400 mM NaCl, 1 mM EDTA pH 8.0, 1 mM EGTA pH 8.0, 1 mM DTT, and 40 μg/mL PMSF) and centrifuged (5 min, 4°C, 16,000 × *g*). After centrifugation, protein concentrations of the supernatant were determined by Bradford assay, and the extract was boiled in Laemmli buffer. Equal amounts of protein were separated with SDS-PAGE. Gels were blotted onto a nitrocellulose membrane, which was blocked afterward in Rotiblock (Carl Roth). After application of the first Ab, an infrared-fluorescent-dye-conjugated secondary Ab (Licor) was used. Signals were detected with an infrared-based laser scanning detection system (Odyssey Classic, Licor) and Licor Image Studio (version 5.2) was used for analysis. Anti-BRG1 (Bethyl Laboratories, catalog no. A303-877 A) and anti-β-actin (Sigma-Aldrich, catalog no. A1978) Abs were used for immunoblotting at 1:1,000 and 1:2,000 dilutions, respectively.

### *LINC00607* mutants, *in vitro* transcription and biotinylation of *LINC00607* constructs, and RNA pulldown assay

The individual pcDNA3.1+LINC00607 deletion constructs were obtained from Biomatik. The plasmids were verified by Sanger sequencing.

For *in vitro* transcription, SmaI-linearized *LINC00607* constructs were purified by DNA-EtOH precipitation, and DNA was subjected to *in vitro* transcription with the HiScribe T7 High Yield RNA Synthesis Kit (NEB). Afterward, DNA was digested with RQ DNase I (Promega). The remaining RNA was purified with the RNeasy Mini Kit (QIAGEN) and biotinylated at the 3′ end with the Pierce RNA 3′ end biotinylation kit (Thermo Fisher).

For the RNA pulldown assay, 50 ng *in vitro* transcribed *LINC00607* RNA was heated for 2 min at 90°C in RNA folding buffer (10 mM Tris pH 7.4, 100 mM KCl, and 10 mM MgCl_2_) and then put on room temperature for 20 min to allow RNA folding. The binding reaction of HUVEC nuclear extract with 50 ng *in vitro* transcribed, biotinylated, and folded *LINC00607* full-length, pcDNA3.1+LINC00607Δexon7 or pcDNA3.1+LINC00607Δexon1–6 RNA was performed in buffer R (20 mM Tris/HCl pH 8.0, 150 mM KCl, 2 mM EDTA pH 8.0, 5 mM MgCl_2_, and 20 U/mL SUPERaseIn RNase Inhibitor [Thermo Fisher Scientific]) for 3 h with a head-over-tail rotator at 4°C. Afterward, equilibrated streptavidin beads were added, and the samples were incubated for 1 h on the head-over-tail rotator at 4°C. Subsequently, the beads were washed three times for 5 min at 4°C with buffer R. Elution and purification of the RNAs was done with TRIzol (Thermo Fisher) followed by reverse transcription and RT-qPCR. For analysis, AriaMX qPCR software version 1.7 (Agilent) was used.

### Overexpression of *LINC00607* mutants

Plasmid overexpression of HUVECs or JEG-3 cells with full-length pcDNA3.1+LINC00607, pcDNA3.1+LINC00607Δexon7, pcDNA3.1+LINC00607Δexon1–4 + 7, pcDNA3.1+LINC00607Δexon5–7, pcDNA3.1+LINC00607Δexon5–6, pcDNA3.1+LINC00607Δexon1–6, or pcDNA3.1+LINC00607Δexon1–4 was performed with the Neon electroporation system (Invitrogen) according to the manufacturer’s instructions. The overexpression was performed for 24 h. After RNA isolation and reverse transcription, RT-qPCR was performed.

### Splice-blocking MOs and ASOs

MOs were purchased as custom oligos from Gene Tools. They had the following sequences: MO e15-5′ (15I/10E), 5′-GTATACAGTTCTAGGTACACAGTAA-3′; MO e14-3′, 5′-AAAAAAGTCTCTTACCAGGCTCTTG-3′; MO e13-3′-i13, 5′-TTTTGTTGCAGTGCTCACCTCTGAT-3′; MO e15-5′ (7I/18E), 5′-ACGTTGATGTATACAGTTCTAGGTA-3′; MO e15-5′ (8I/7E), 5′-TACAGTTCTAGGTAC-3′; MO e15-5′ (21I/4E), 5′-AGTTCTAGGTACACAGTAAATAATC-3′; MO e15-5′ (25I/0E), 5′-CTAGGTACACAGTAAATAATCATAT-3′; and MO e16-5′, 5′-GTCCGAGGTTCCTGGAGAGAAAAAA-3′. As negative control (CTL), the CTL MO PCO-StandardControl-100 (Gene Tools) with the sequence 5′-CCTCTTACCTCAGTTACAATTTATA-3′ was used.

For RNA streptavidin pulldown assays, the 3′ end biotinylated MO e15-5′, e14, and e13-3′-i13 were used (purchased as custom oligos from Gene Tools), and the pulldown assay was performed as described above.

ASOs were purchased as custom oligos from Merck. They had the following sequences: ASO e15-5′ (15I/10E), 5′-GTATACAGTTCTAGGTACACAGTAA-3′; ASO e15-5′ (7I/18E), 5′-ACGTTGATGTATACAGTTCTAGGTA-3′; ASO e15-5′ (20I/15E), 5′-TTGATGTATACAGTTCTAGGTACACAGTAAATAAT-3′; ASO e15-5′ (25I/25E), 5′-GATGGTGACGTTGATGTATACAGTTCTAGGTACACAGTAAATAATCATAT-3′; ASO e15-5′ (25I/0E), 5′-CTAGGTACACAGTAAATAATCATAT-3′; and ASO e15-5′ΔAS, 5′-GTATACAGGGGGAGGTACACAGTAA-3′. As negative CTL, a negative CTL antisense oligo with the sequence 5′-CCTCTTACCTCAGTTACAATTTATA-3′ (Merck) was used.

MOs (10 μM) or ASOs (1 μM) were electroporated in E2 buffer in HUVECs with the Neon electroporation system (Thermo Fisher Scientific) (1,400 V, 1 × 30-ms pulse) according to the manufacturer’s instructions. The overexpression was performed for 24 h.

### RNAscope

RNA *in situ* hybridization was performed on tissue sections using RNAscope 2.5 HD Detection Reagents and the HybEZ system (Advanced Cell Diagnostics [ACD], catalog no. 322310). The single-plex chromogenic brown assay was used according to the manufacturer’s instructions, with minor changes. Paraffin-embedded, formalin-fixed sections were deparaffinized prior to staining. Sections were treated with hydrogen peroxide before incubation in boiling target retrieval buffer for 20 min using a Braun steamer and dried. Sections were incubated with protease plus reagent for 30 min in a HybEZ oven at 40°C in a humidified atmosphere. After washing twice in 1× wash buffer, RNAscope probes were added, and the sections were incubated in the HybEZ oven for 2 h. The probe (*LINC00607*, ACD, catalog no. 894351; *HLA-G*, ACD, catalog no. 429941; DapB, ACD, catalog no. 310043) signal was amplified in a series of amplification and washing steps. Amplification steps 1–4 were performed in the HybEZ oven at 40°C, and steps 5 and 6 were performed at room temperature. The first and third steps were incubated for 30 min, whereas the second, fourth, and sixth steps were incubated for 15 min. Amplification step five was incubated for 45 min. After the last amplification step, signals were detected by incubating 3,3′-diaminobenzidine for 20 min at room temperature. The sections were dehydrated and mounted using EcoMount. Images were acquired using a light microscope.

### RNA IP

To identify RNAs bound to a protein of interest, specific antibodies (Abs) and protein A-coated beads were used to immunoprecipitate the target protein before RT-qPCR or RNA-seq to identify RNAs. Cells were grown to 80% confluence on a 10-cm plate (roughly 3 million cells) and washed once with Hank’s buffer. We added 6 mL Hank’s buffer to the cells on ice and irradiated with 0.150 J/cm^2^ 254-nm UV light. Cells were scraped twice in 500 μL Hank’s buffer and centrifuged at 1,000 × *g* at 4°C for 4 min. Isolation and lysis of the nucleus was performed as outlined above for protein isolation. We took 10% of the nuclear lysate as the “input” sample for normalization of the IP pulldown samples. We pre-coupled 4 μg Ab to 50 μL protein A magnetic beads in buffer C for 1 h at room temperature and then washed once with high-salt buffer and twice with buffer C3. The Ab-coupled beads were added to the nuclear lysate and rotated for 16 h at 4°C.

Samples were placed on a magnetic bar and the lysate discarded. In RNA immunoprecipitation (RIP) experiments without stringent washes, the beads were washed three times in high-salt buffer (50 mM Tris-HCl, 1 M NaCl, 1 mM EDTA, 0.1% SDS, 0.5% sodium deoxycholate, 1% NP-40, 1 mM DTT, and 40 μg/mL PMSF) at 4°C for 10 min per wash. Beads were then washed twice in bead wash buffer 2 (20 mM TrisHCl, 10 mM MgCl_2_, 0.2% Tween, 1 mM DTT, and 40 μg/mL PMSF). For the elution of RNA, the remaining wash buffer was removed, and 1 mL QIAzol (QIAGEN) was added to the beads and incubated at room temperature for 10 min. We added 400 μL chloroform to the samples and vortexed for 10 s, followed by incubation for a further 10 min at room temperature. Samples were then centrifuged at 12,000 × *g* for 15 min at 4°C. We transferred 500 μL of the upper aqueous phase to a new tube and added 2 μL glycogen (GlycoBlue Coprecipitant, Thermo Fisher, catalog no. AM9515) and 500 μL isopropanol. Samples were inverted multiple times and incubated at room temperature for 10 min before being centrifuged again at 12,000 × *g* for 10 min. The supernatant was removed and the pellet washed with 1 mL 75% ethanol by vortexing. The pellet was centrifuged at 7,500 × *g* for 5 min at 4°C, dried, and resuspended in 30 μL nuclease-free water. RNA samples were reverse transcribed for qPCR as described above.

For RIP experiments with stringent washes, the experiment was done as described above, except that the beads were washed after incubation with nuclear lysate with the covalent linkage and affinity purification (CLAP) wash buffers 1–5 (NLS CLAP wash buffer, 1 M NaCl CLAP wash buffer, 8 M urea CLAP wash buffer, Tween 20 CLAP wash buffer, TEV CLAP wash buffer), three times each at 90°C for 3 min, as described in Guo et al.^60^

### JunctionSeq

As input to perform splicing analysis, our publicly available RNA-seq after lentiCRISPRv2-mediated KO of *LINC00607* in HUVEC from Boos et al.^34^ was used. Splicing analysis and visualization was performed by counting exons and junctions by Qorts (version 1.3.6) followed by analysis in R with the *JunctionSeq* package (version 1.8.0) using false discovery rate (FDR) significance cutoff at 0.05.^35^ For visualization of Figure 1C, geometric mean values of the absolute log_2_ fold change of the maximally up- and downregulated exon per gene were used, as well as the geometric mean of the negative log_10_ of the adjusted *p* value.

### eQTL analysis

We performed eQTL mapping for ENST00000445174 (*LINC00607*), ENST00000541932 (*sFLT1*), and ENST00000282397 (*FLT1*) transcripts using the Matrix eQTL R package (version 2.3).^61^ SNPs located within 500 kb upstream of the TSS and 500 kb downstream of the transcription end sites of the annotated genes were included in the analysis. Transcript expression levels in transcripts per million from 11 tissue types (aorta, coronary artery, tibial artery, subcutaneous adipose tissue, visceral [omentum] adipose tissue, heart atrial appendage and left ventricle, liver, spleen, skeletal muscle, and blood) and SNP genotypes were obtained from the Genotype-Tissue Expression (GTEx) V8 project.^62^ A linear regression model was applied to test SNP-expression pairs, and eQTLs were considered significant at an FDR threshold of 0.05.

### Identification of enriched RNA binding protein motifs in RNA-seq

For the identification of putative RNA binding protein motifs in the sequence of *LINC00607*, the sequence of exon 7 was used as input to the R package *universalmotif* (version 1.24.2),^63^ which was used for motif scanning. The RNA binding protein motifs used were taken from the latest update of the CISBP-RNA database (https://cisbp-rna.ccbr.utoronto.ca/, updated May 7, 2025),^38^ and only human motifs were considered. Motif scanning results were assigned a *p* value that were subsequently adjusted for multiple testing, resulting in a final *q* value per motif hit.

### Apoptosis assay

After electroporation of the MO e15-5′ or control MO, 4,000 HUVECs were seeded on a 96-well plate with 200 μL EGM. Incucyte Annexin V Green dye (dilution 1:500 in EGM) (Sartorius, catalog no. 4642) was used to stain apoptosis-positive cells. Observation was performed for 141 h in an Incucyte live-cell analysis system (Sartorius), which imaged the cells every 3 h and subsequently counted the apoptosis-positive cells in all images. Counts per image were analyzed with Incucyte 2022B Rev2 (Sartorius).

### sFLT 1 ELISA

Soluble FLT1 (sVEGFR1) levels were quantified in HUVEC-conditioned medium using the Human sVEGF-R1 ELISA Kit (Invitrogen, catalog no. BMS268-3) according to the manufacturer’s instructions. Briefly, HUVECs were subjected to siRNA-mediated knockdown of *LINC00607* (or Scr CTL) for 72 h, followed by electroporation of MO e15-5′ or the MO negative CTL (10 μM, 24 h). Cell culture supernatants were then collected, cleared by brief centrifugation to remove debris, and analyzed by ELISA. Absorbance was measured on a Tecan infinite M200 Pro plate reader, and sFLT1 concentrations were measured with Tecan i-control (version 3.7.3.0) and calculated from a standard curve generated with the kit standards.

### Other bioinformatic tools used

The secondary structure of the exon was predicted with the Vienna RNA fold prediction tool,^64^ and for visualization, the forna view RNA secondary structure visualization tool^65^ was used.

GO enrichment analysis (powered by PANTHER) of the high-confidence isoform-switch candidates was performed by using the web interface of geneontology.com with standard parameters.^66^^,^^67^^,^^68^ For GO biological process complete, the GO database released July 22, 2025 was used. For Reactome pathways, Reactome version 86 (released September 7, 2023) was used.

### Statistical analysis

Unless otherwise indicated, data are given as means ± standard deviations (SDs). Calculations were performed with GraphPad Prism 10 or BiAS.10.12. The latter was also used to test for normal distribution and similarity of variance. In cases of multiple testing, the Bonferroni correction was applied. For multiple group comparisons, ANOVA followed by post hoc testing was performed. Individual statistics of dependent samples were performed by paired *t* test, of unpaired samples by unpaired *t* test, and if not normally distributed by Mann-Whitney test. *p* values <0.05 was considered significant. Unless otherwise indicated, *n* indicates the number of individual experiments.

## Data and code availability

RNA-seq of *LINC00607* KO and non-target control was published by Boos et al. in 2023^34^ and is publicly available at NCBI GEO with the accession number GSE199878. Other publicly available datasets used in this study are placental cell types single-cell RNA-seq dataset (Vento-Tormo et al.^40^), placental gene expression available at NCBI GEO with the accession number GSE182381,^43^ placental tissue available at NCBI GEO with the accession numberGSE256412,^44^ and induced pluripotent stem cell modeling MSC, EVT naive, and EVT prime available at NCBI GEO with the accession number GSE243579.^45^

## Acknowledgments

The authors thank Katalin Palfi and Alexandra Friemel for excellent technical assistance. This work was supported by the 10.13039/501100005687Goethe University Frankfurt am Main; the 10.13039/501100001659DFG Excellence Cluster 10.13039/501100021703Cardiopulmonary Institute (10.13039/501100021703CPI) EXS2026 (project no. 390649896, to R.P.B.); the 10.13039/501100001659DFG Transregio TRR267 (project ID 403584255-TRR 267), TP A03 (to M.M.-M.), TP A04 (to M.S.L.), TP A06 (to R.P.B.), TP B04 (to R.A.B.), TP B05 (to Z.C.), and TP Z02 (to I.W.); the 10.13039/100010447German Centre for Cardiovascular Research (10.13039/100010447DZHK, Standortproject Rhine Main and trinational project ReGenLnc, project no. 81X2200165); the 10.13039/501100000274British Heart Foundation (grant no. SP/F/22/150029, ReGenLnc, to A.H.B.); the Dutch Heart Foundation (grant no. 02-001-2021-B019, ReGenLnc, to R.A.B.); the Amandus und Barbara Pauli-Stiftung (to J.A.O.); and the Dr. Rolf Schwiete-Stiftung.

## Author contributions

F. Lam, T. Warwick, J.A.O., N.-N.K., F. Louwen, and M.S.L. designed the experiments. F. Lam, T. Warwick, J.A.O., A.Y.K., J.I.P., P.T., M.E.B., S.G., N.-N.K., and M.S.L. performed the experiments. F. Lam, T. Warwick, J.A.O., S.G., N.-N.K., and M.S.L. analyzed the data. T. Warwick, J.A.O., A.D., S.G., and Z.C. performed the bioinformatics. O.N., R.D., I.W., T. Walther, R.A.B., A.H.B., M.M.-M., F. Louwen, and R.B.P. contributed scientific input and critical discussion. F. Lam, R.B.P., and M.S.L. wrote the manuscript. All authors interpreted the data and approved the manuscript.

## Declaration of interests

The Goethe University Frankfurt has filed a patent application related to this work, on which M.S.L. and R.P.B. are listed as inventors (file no. EP25218896, submission no. EP25218896.6).

## Supplemental information

Document S1. Figures S1–S4

Table S1. *JunctionSeq* differentially spliced exons in endothelial cells after *LINC00607* KO

Table S2. BRG1 iCLIP-binding site gene scores for snRNAs

Table S3. eQTLs shared *LINC00607* (ENST00000445174) sFLT1 (ENST00000541932)

Table S4. eQTLs shared *LINC00607* (ENST00000445174) FLT1 (ENST00000282397)

Document S2. Article plus supplemental information
