# Supplementary material for: LINC00607 facilitates endothelial VEGF-A receptor FLT1 splicing
Source: Mol Ther. 2026 Apr 4;34(7):4323–38. doi: 10.1016/j.ymthe.2026.03.038 (PMC13330067; doi:10.1016/j.ymthe.2026.03.038)
Supplement: Supplementary file 1 [file mmc1.pdf]

YMTHE, Volume *34*

Supplemental Information

LINC00607 facilitates endothelial VEGF-A receptor FLT1 splicing

Frederike Lam, Timothy Warwick, James A. Oo, Agnes Y. Krüger, Nina-Naomi Kreis, Anastasiia Diagel, Judit Izquierdo Ponce, Praveenya Tirunagari, Marie E. Bayer, Olivia Nonn, Ralf Dechend, Thomas Walther, Reinier A. Boon, Andrew H. Baker, Stefan Günther, Ilka Wittig, Zhifen Chen, Michaela Müller-McNicoll, Frank Louwen, Ralf P. Brandes, and Matthias S. Leisegang

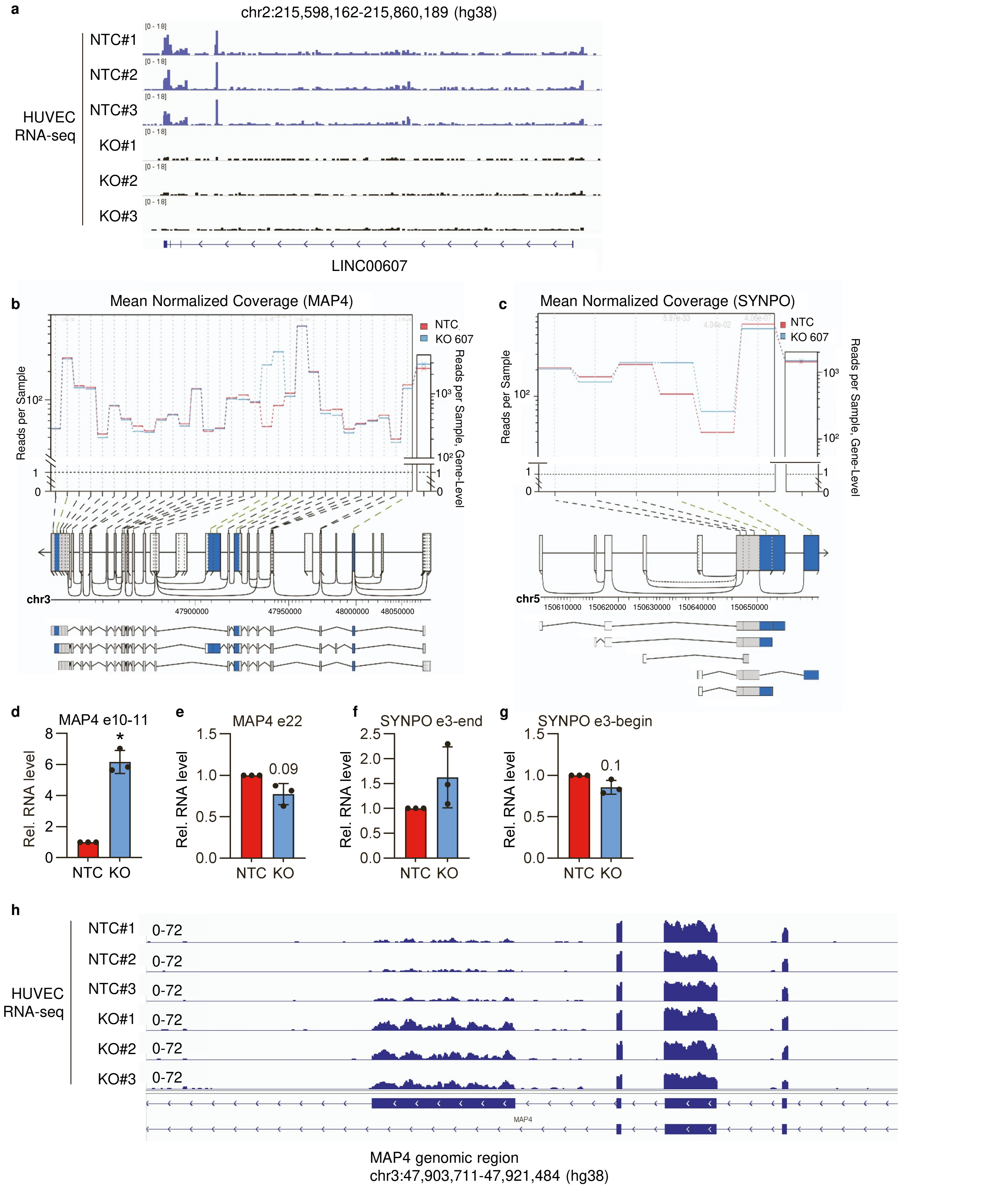

Figure S1

a IGV browser traces of RNA-seq in HUVEC with NTC and *LINC00607* KO the locus chr2:215,598,162-215,860,189 (hg38), which harbors *LINC00607*. n=3. **b, c** Gene plot profiles of *MAP4* (b) and *SNYPO* (c) from *JunctionSeq* analysis. Upper central plotting area displays mean normalized read counts of each exon in *LINC00607* KO (blue) and non-target control (NTC, red) and the gene-level normalized read counts on the right. Displayed below is the genomic locus, its exon-intron structure and a representation of isoforms. Indicated in blue are exons and introns overrepresented in *LINC00607* KO reads. **d-g** Relative expression of diff. spliced transcripts by RT-qPCR using exon- and/or intron-spanning primers for *MAP4* exon 10-11 (alternative exon) (d), *MAP4* exon 22 (e), *SYNPO* exon 3 (end) (f) and *SYNPO* exon 3 (begin) (g). Paired t-test. n=3. **h** IGV browser traces of RNA-seq in HUVEC with NTC and *LINC00607* KO displaying alternative exon 10 inclusion. Error bars are defined as mean +/- SD. *p<0.05.

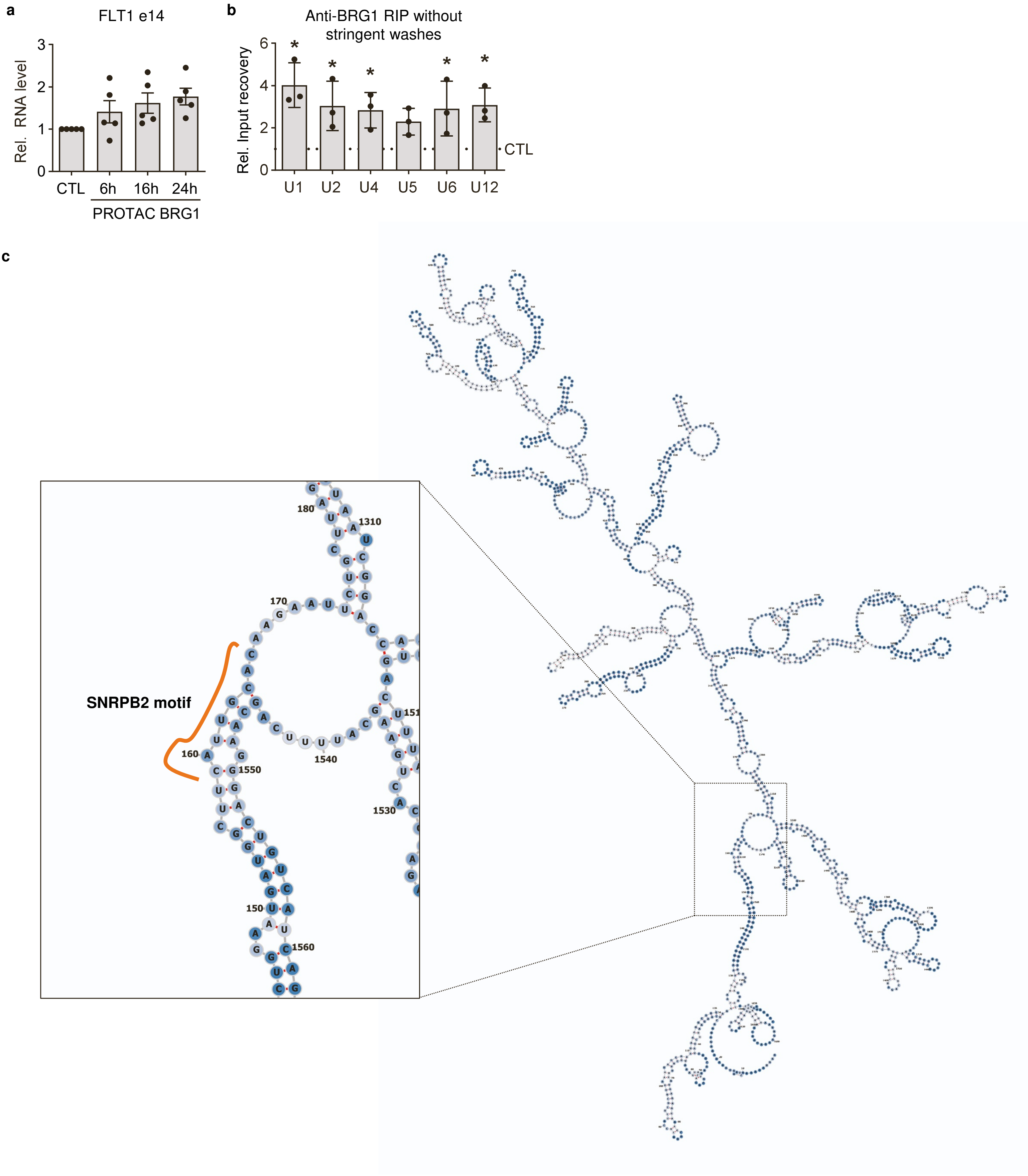

Figure S2

a, RT-qPCR of FLT1 e14 after treatment of HUVECs with the BRG1 PROTAC AU-15330 (1 µM, 6-24 h). n=5, paired t-test. **b,** Anti-BRG1 RNA immunoprecipitation (without stringent washes) followed by RT-qPCR for individual U snRNAs. Rel. input recovery of the respective U snRNA is shown. IgG served as negative control (CTL) and was set to 1. n=3, One-Way ANOVA with Bonferroni post-hoc test. **c**, *LINC00607* exon 7 RNA minimum free energy (MFE) structure drawing encoding base-pair probabilities done with the Vienna RNA fold prediction tool. The *forna* view RNA secondary structure visualization tool was used to visualize the secondary structure. The orange line indicates the RBP motif of SNRPB2. Error bars are defined as mean +/- SD. *p<0.05.

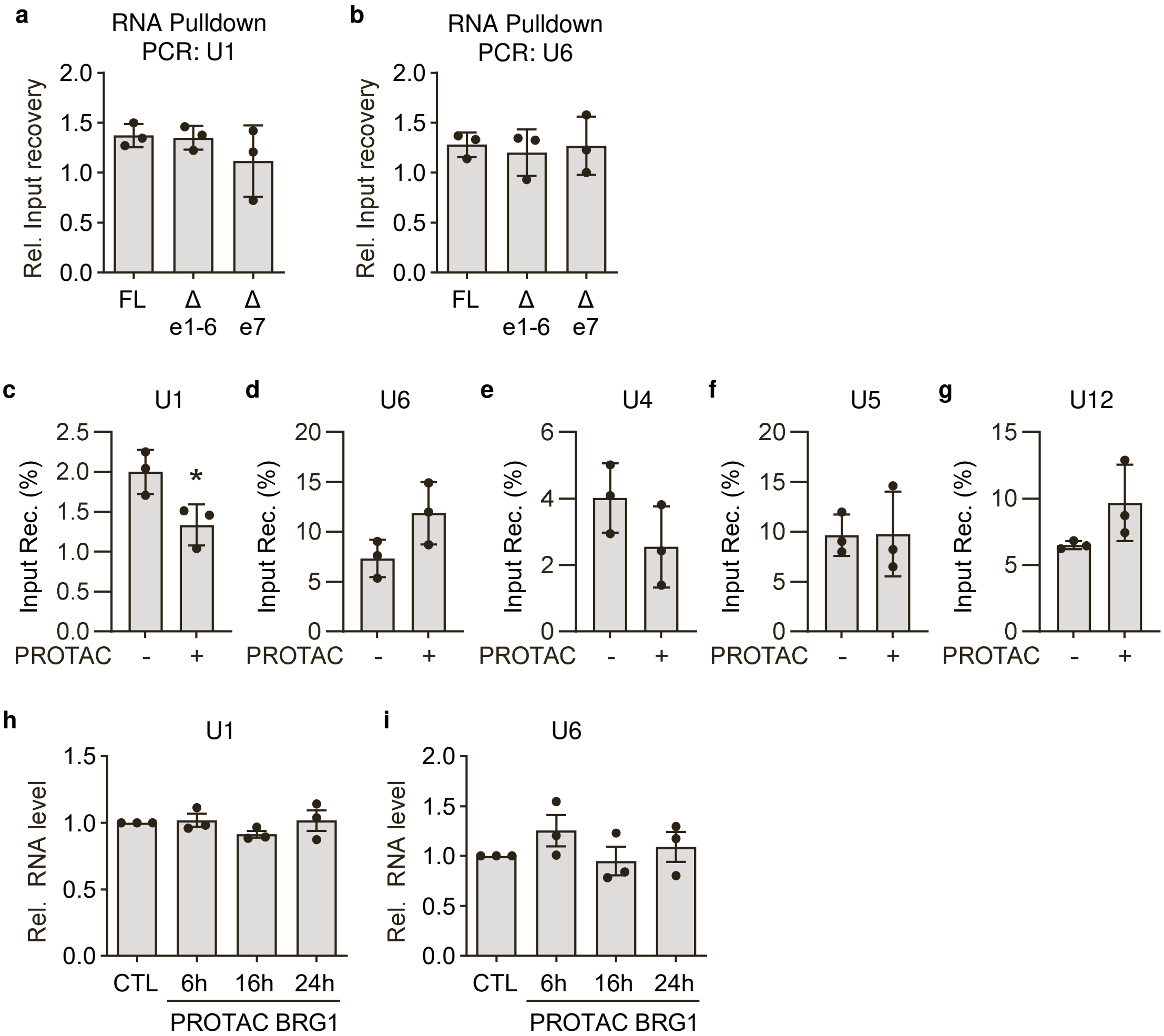

Figure S3

a, b, RNA pulldown assay of *in vitro* transcribed and biotinylated *LINC00607* full length (FL) or deletion mutants. RT-qPCR for U1 snRNA (a) and U6 snRNA (b) is shown. n=3, One-Way ANOVA with Bonferroni post-hoc test. **c-g**, RNA pulldown assay of *in vitro* transcribed and biotinylated *LINC00607* full length after stimulation of cells with the BRG1 PROTAC for 16 h. RT-qPCR U1 (c), U6 (d), U4 (e), U5 (f), U12 (g) snRNA is shown. n=3, paired t-test. **h**, **i**, RT-qPCR of U1 snRNA (h) and U6 snRNA (i) after treatment of HUVECs with the BRG1 PROTAC AU-15330 (1 µM, 6-24 h). n=3, paired t-test. Error bars are defined as mean +/- SD. *$p<0.05$.

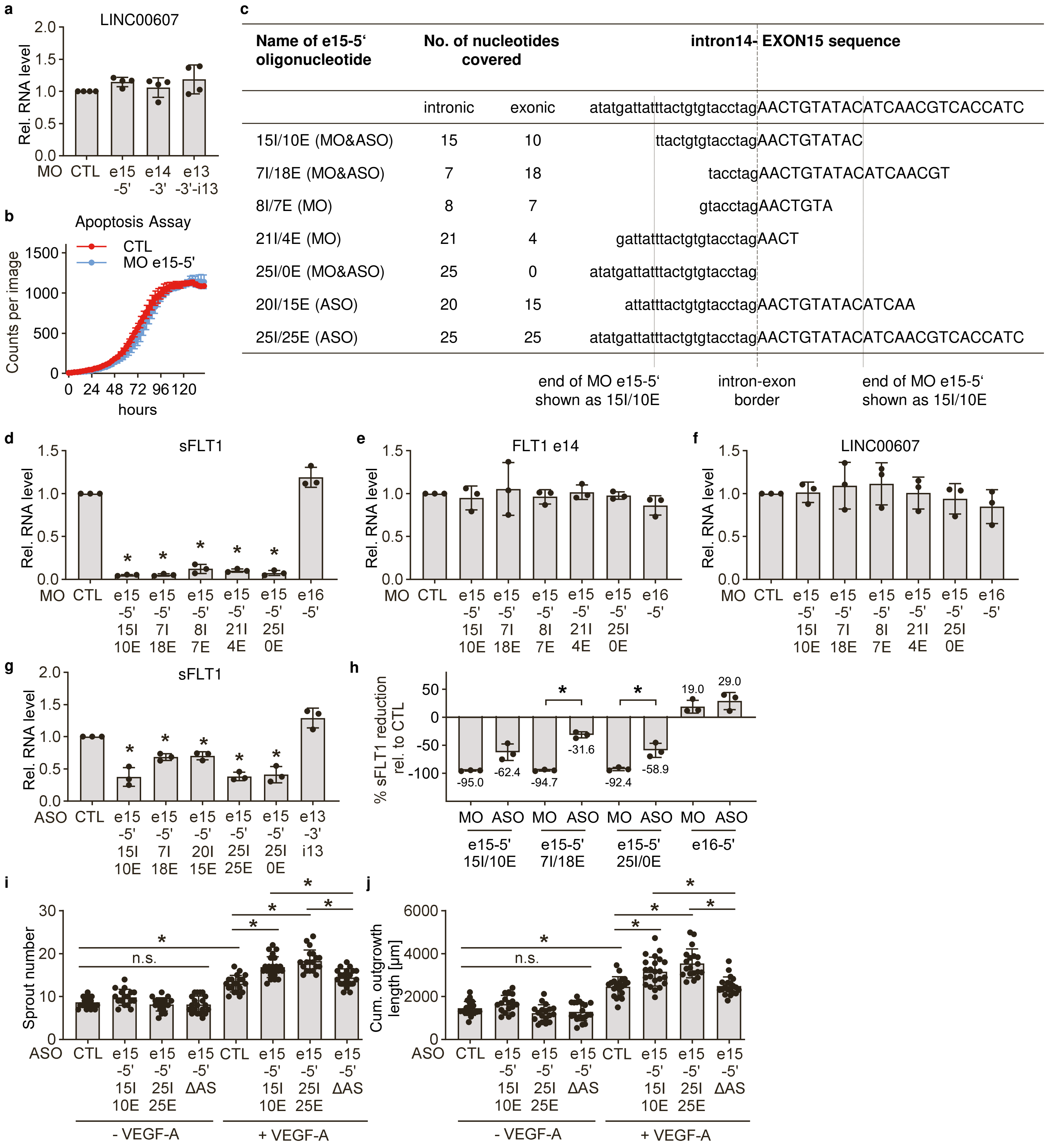

Name of e15-5‘ oligonucleotide	No. of nucleotides covered		intron14- EXON15 sequence
	intronic	exonic	atatgattatttactgtgtacctagAACTGTATACATCAACGTCACCATC
15I/10E (MO&ASO)	15	10	ttactgtgtacctagAACTGTATAC
7I/18E (MO&ASO)	7	18	tacctagAACTGTATACATCAACGT
8I/7E (MO)	8	7	gtacctagAACTGTA
21I/4E (MO)	21	4	gattatttactgtgtacctagAACT
25I/0E (MO&ASO)	25	0	atatgattatttactgtgtacctag
20I/15E (ASO)	20	15	attatttactgtgtacctagAACTGTATACATCAA
25I/25E (ASO)	25	25	atatgattatttactgtgtacctagAACTGTATACATCAACGTCACCATC

Figure S4

a, RT-qPCR of *LINC00607* after electroporation of HUVECs with the indicated splice-blocking morpholinos (10 µM, 24h). Data is normalized to β-Actin. n=4, paired t-test. **b**, Apoptosis assay of HUVECs electroporated with the MO e15-5'. HUVECs were treated with an apoptosis marker and observed for a further 141h in an Incucyte system. Cells were imaged every 3h and subsequently the apoptosis-positive cells were counted. Red indicates the control MO, blue indicates the e15-5' MO. **c,** Splice-blocking MOs and antisense oligonucleotides (ASOs) of various lengths and different intron/exon compositions used. These vary specifically regarding the target sequences in the region of the intron/exon border (border, indicated by a dashed middle line) and extend up to a maximum of 25 nt into the intron (I) and 25 nt into the exon (E). The sequence of the intron region is shown in lowercase letters, and the sequence of the exon in uppercase letters. The dotted lines indicate the region targeted by the originally used e15-5' MO (=15I/10E, i.e., 15 nt in the intron, 10 nt in the exon). **d-f**, RT-qPCR of sFLT1 (d), FLT1 e14 (e) or *LINC00607* (f) of HUVECs treated with the indicated MOs (10 µM). The splice-blocking MO targeting the downstream exon 16 (e16-5') remains ineffective, as it does not target the intron14-exon15 border region. MOs used were not coupled to biotin at the 3'end, indicating that biotin is not required. Data is normalized to β-Actin. n=3, One-Way ANOVA with Dunnett post-hoc test. **g**, RT-qPCR of sFLT1 of HUVECs treated with various ASO (1 µM, 24h). I, number of nt matching the intronic site next to the splice site; E, number of nt matching the exonic site next to the splice site. n=3, One-Way ANOVA with Dunnet post-hoc test. *, p<0.05. **h**, Quantitative comparison of MOs vs. ASOs of sFLT1 RNA levels. n=3, One-Way ANOVA with Tukey post-hoc test. **i-j**, Quantification of spheroid outgrowth assays after electroporation of HUVECs (1 µM, 24h) with various ASOs. Sprout number (i) and cumulative outgrowth length (j) were quantified. I, number of nt matching the intronic site next to the splice site; E, number of nt matching the exonic site next to the splice site. In ASO e15-5' ΔAS, the MO sequence was changed from TTCT (AGAA on pre-mRNA) to GGGG. Error bars are mean +/- SD. n.s., not significant. *p<0.05.

Supplemental Tables

Table S1. JunctionSeq differentially spliced exons in endothelial cells after LINC00607 KO.
Available as a separate Excel file.

Table S2. BRG1 iCLIP-Binding Site Gene Scores for snRNAs.
Available as a separate Excel file.

Table S3. eQTLs shared LINC00607(ENST00000445174) sFLT1(ENST00000541932).
Available as a separate Excel file.

Table S4. eQTLs shared LINC00607(ENST00000445174) FLT1(ENST00000282397).
Available as a separate Excel file.
